# Contrasting Responses of Plastid Terminal Oxidase Activity Under Salt Stress in Two C_4_ Species With Different Salt Tolerance

**DOI:** 10.3389/fpls.2020.01009

**Published:** 2020-07-07

**Authors:** Jemaa Essemine, Ming-Ju Amy Lyu, Mingnan Qu, Shahnaz Perveen, Naveed Khan, Qingfeng Song, Genyun Chen, Xin-Guang Zhu

**Affiliations:** National Key Laboratory of Plant Molecular Genetics, CAS Center for Excellence in Molecular Plant Sciences, Institute of Plant Physiology and Ecology, Shanghai Institute for Biological Sciences, Chinese Academy of Sciences, Shanghai, China

**Keywords:** *Setaria viridis*, *Spartina alterniflora*, C_4_ species, salt stress, photoprotective mechanisms, PTOX

## Abstract

The present study reveals contrasting responses of photosynthesis to salt stress in two C_4_ species: a glycophyte *Setaria viridis* (*SV*) and a halophyte *Spartina alterniflora* (*SA*). Specifically, the effect of short-term salt stress treatment on the photosynthetic CO_2_ uptake and electron transport were investigated in *SV* and its salt-tolerant close relative *SA*. In this experiment, at the beginning, plants were grown in soil then were exposed to salt stress under hydroponic conditions for two weeks. *SV* demonstrated a much higher susceptibility to salt stress than *SA*; while, *SV* was incapable to survive subjected to about 100 mM, *SA* can tolerate salt concentrations up to 550 mM with slight effect on photosynthetic CO_2_ uptake rates and electrons transport chain conductance (*g_ETC_*). Regardless the oxygen concentration used, our results show an enhancement in the P_700_ oxidation with increasing O_2_ concentration for SV following NaCl treatment and almost no change for SA. We also observed an activation of the cyclic NDH-dependent pathway in *SV* by about 2.36 times upon exposure to 50 mM NaCl for 12 days (d); however, its activity in *SA* drops by about 25% compared to the control without salt treatment. Using PTOX inhibitor (*n-PG*) and that of the Q_o_-binding site of Cytb_6_/f (DBMIB), at two O_2_ levels (2 and 21%), to restrict electrons flow towards PSI, we successfully revealed the presence of a possible PTOX activity under salt stress for *SA* but not for *SV*. However, by q-PCR and western-blot analysis, we showed an increase in PTOX amount by about 3–4 times for *SA* under salt stress but not or very less for *SV*. Overall, this study provides strong proof for the existence of PTOX as an alternative electron pathway in C_4_ species (*SA*), which might play more than a photoprotective role under salt stress.

## Introduction

Soil salinity constitutes a major environmental scourge that adversely affects crop productivity and yield quality (Horie and Schroeder, 2004). Approximately one fifth of the world’s cultivated area and about half of the world’s irrigated lands are affected by the salinity constraint (Sairam and Tyagi, 2004). Mechanisms of how plants respond and/or tolerate salt stress are under intensive study (Zhu, 2001; Munns and Tester, 2008). To survive and overcome salt stress, plants mostly respond and acclimate with complex mechanisms including morphological, physiological and biochemical strategies (Taji et al., 2004; Acosta-Motos et al., 2015), which serve to modulate ion homeostasis, compatible compounds biosynthesis, sequestration of toxic ions, and reactive oxygen species (ROS) scavenging systems (Stepien and Klobus, 2005; Flowers and Colmer, 2008; Stepien and Johnson, 2009). In this study, we report that a protein involved in alternative electron transfer, PTOX, might be related to salt tolerance in C_4_ plants.

The protein PTOX is a plastid-localized protein involved in the plastoquinol oxygen oxido-reductase electrons flow process. PTOXwas discovered in the so-called immutans of *Arabidopsis* which shows a variegated leaf phenotype (Rédei, 1963; Wetzel et al., 1994; Carol et al., 1999; Wu et al., 1999; Shahbazi et al., 2007). In chloroplasts, PTOX is situated at the stroma lamellae (SL) directly exposed to the stroma compartment (Lennon et al., 2003) and it is essential for the plastid development and carotenoid biosynthesis in plants (Carol et al., 1999; Aluru et al., 2001). PTOX is also involved in photosynthetic electron transport (Okegawa et al., 2010; Trouillard et al., 2012), chlororespiration (Cournac et al., 2000), poising chloroplast redox potential under dark (Nawrocki et al., 2015), and in response to abiotic stress (McDonald et al., 2011; Sun and Wen, 2011). It has been reported that plants grown under moderate light and non-stressful conditions exhibit low PTOX levels (uniquely 1 PTOX for 100 PSII photosystem; Lennon et al., 2003); in contrast, high PTOX levels have been characterized for plants exposed to various abiotic stresses such as heat, high light and drought (Quiles, 2006), high soil salinity (Stepien and Johnson, 2009), cold treatment and high intensities of visible light (Ivanov et al., 2012) and UV light (Laureau et al., 2013).

In this study, in an effort to understand potential mechanism of how the halophyte *SA* tolerate high salt stress, we show that compared to a glycophyte species *SV*, under high salt stress (500 mM), *SA* showed increased expression of PTOX, which might have played a critical role for the maintenance of photosynthetic physiology and hence high photosynthetic efficiency of this species under salt constraint.

## Materials and Methods

### Plant Material

Seeds of *SA* were collected from *San-San* Lake in South East Shanghai city at the end of November in 2015 and 2016. The cleaned spikelets were stored in wet tissue (cloth) in sealed plastic at 4°C in the refrigerator. *SA* mature seeds require two to three months, after-ripening, wet storage in cold (stratification) to break dormancy (Garbisch and McIninch, 1992) and they remain viable for about one year. Seeds of *SV* were rinsed several times with tap water and then transferred to Petri-dishes and covered with water till germinate. After germination, they were transferred into potted soil. When the young seedlings of *SA* were about 2 cm in length and started greening, they were removed from the glass petri dishes. Trays containing *SA* seedlings were kept indoor at a temperature between 25 and 27°C, under fluorescent light at a photosynthetic photon flux density (PPFD) of 80–120 μmol m^−2^ s^−1^ with a photoperiod of 16/8 h for light/dark, respectively. Two-month old healthy plants with large expanded leaves where transferred to a hydroponic system for salt treatment. For *SV*, dry seeds were directly sown into wet potted soil, which was maintained wet by spraying water daily on the soil till the seeds germinated. *SV* grew under the same photoperiod and temperature conditions as *SA*. Nutrient was added routinely to ensure healthy growing plants before transferring them to hydroponic medium for salt stress treatment.

### Salt Stress (NaCl) Treatment

Salt (NaCl) treatment was applied to hydroponic solution. During the plants transfer, roots were washed adequately with tap water then rinsed with deionized water. Four-week-old *SV* and 10-week-old *SA* plants were treated with 0, 50, 100, 250, 400, and 550 mM NaCl for up to 15 d. The composition of the hydroponic medium was as described by Hoagland and Arnon (1950).

### Determination of Monovalent Cations (Na^+^ and K^+^) Content

Leaves were harvested and washed with deionized water twice. Then, the leaf samples were dried in the oven firstly at around 105°C for 2 h, subsequently at 65–70°C for 72 h, and then weighed to record their dry weights. Lyophilized leaves were ground to powder using pestle and mortar for mineral nutrient evaluation. The grinded samples to powder were thereafter dissolved in 10 ml of HNO_3_ (0.1N) for 60 min at 95°C to extracted the major cations. The obtained solutions were subsequently filtered through Whatman filter paper, diluted with deionized water and processed for Na^+^ and K^+^ determination. The cations (Na^+^ and K^+^) levels were determined with an atomic spectrophotometer (PerkinElmer, PE AAS 900 F).

### Chlorophyll (Chl) Content Measurements

The total Chl content was determined as previously described by Porra et al. (1989). Leaf segments (0.1 g) were first washed with distilled water and then kept in 1 ml acetone (80%) at 4°C for 12 d. Then samples were centrifuged at high speed (13,000*g*) for 5 min and subsequently their absorbance was measured at 663 and 645 nm using a UV visible spectrophotometer (50 Bio Varian, Varian Inc., Walnut Creek, CA). The total Chl content was determined as follows: total Chl (mg·l^−1^) = (8.02 × OD_663_) + (20.21 × OD_645_), where OD stands for optical density. The results of the Chl content were expressed as mg per gram fresh weight (mg g^−1^ fresh weight, FW) and calculated based on the extinction coefficients and the equations given by Porra et al. (1989).

### Assessment of Photosystem II (PSII) Parameters

PSII efficiency was assessed using the Chl *a* fluorescence induction (FI) technique. We used the multifunctional plant efficiency analyzer (M-PEA; Hansatech, King Lynn, Norfolk, UK) for the evaluation of PSII parameters as reported in details by Essemine et al. (2017). Plants were dark-adapted for at least 1 h at 25°C before measurements. Then, healthy and fully expanded leaves were exposed to saturating orange-red (625 nm) actinic light (AL, 5,000 µmol m^−2^ s^−1^) provided by the LED for 1 second. The ratio of variable fluorescence level F_v_ (F_m_–F_0_) to the maximum fluorescence level F_m_ (F_v_/F_m_) was considered herein to estimate the maximum PSII efficiency. F_m_ (P-level) is the maximum yield of Chl *a* fluorescence and F_0_ (O-level) represents its (Chl *a* fluorescence) minimum (the intensity of Chl *a* fluorescence of dark-adapted leaf with a measuring light of negligible AL intensity). F_v_/F_0_ parameter represents the functional reaction center of PSII. All the parameters listed in the **Table S1** were calculated from the original OJIP curves based on the so-called JIP-test (Strasser et al., 2004). The OJIP curve represents the transient fast chlorophyll *a* fluorescence induction of the dark adapted leaf following excitation with 1 s of saturating orange-red light (625 nm; 5,000_μmol m^−2^ s^−1^; Essemine et al., 2017).

### Setting of PAM Together With Infrared Gas Analyzer to Control CO_2_ and O_2_ Supply

**Figure 1:**
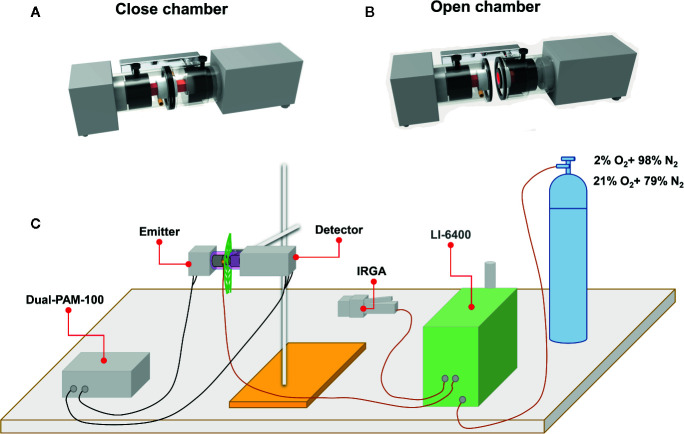
Schematic representation showing the experimental setting of Li-cor 6400 together with Dual-PAM-100, through a chamber holding on the emitter-detector system of the PAM, for controlling CO_2_ level (390 or 2,000 μl L^−^^1^) and using an oxygen source equipped with an oxymeter to adjust oxygen flux from the appropriate O2 source (2 or 21%) to the chamber and accordingly be able to monitor the CO_2_ and O_2_ concentrations in the chamber during measurements. **(A)** Close chamber; **(B)** Open chamber and **(C)** Setting of Li-cor 6400 together with Dual-PAM-100 to control the gas concentration (O_2_ and CO_2_) in the newly designed chamber. See also video for setting. IRGA and PAM mean infrared gas analyzer and pulse amplitude modulation, respectively.

A special chamber was custom-designed and developed to enable precise control of CO_2_ and O_2_ environments. This chamber was tightly mounted on the detector-emitter of the Dual-PAM-100 fluorimeter which was connected through a hole to the Li-COR 6400 portable infrared gas analyzer (IRGA) to control CO_2_ supplies (390 or 2,000 μl L^−1^) by Li-cor and *via* another window to an oxygen source equipped with an oxymeter to adjust the flow of oxygen from the source to the chamber. Oxygen sources with different concentrations (e.g. 2 and 21% as used in this study) are supplied by a gases distribution station (GDS). The setting for experiments using different levels of CO_2_ and O_2_ was as depicted in **Figure 1** and video in **Supplemental Data**.

### Evaluation of P_700_ Redox State in Leaves of *SV* and *SA*

In order to probe the photosynthetic electron flow through PSI during steady-state photosynthesis *in vivo*, we preceded to determine the P_700_ redox state in the light by measuring the oxidation of P_700_ within the leaf as absorbance changes at 830 minus 875 nm to avoid any oxidation of plastocyanin (Pc). P_700_ was oxidized to P_700_^+^ at different intensities of AL ranging from 0 to 1,804 μmol m^−2^ s^−1^ (ΔA) then re-reduced in the dark and finally oxidized to a maximum level of P_700_^+^ under far-red illumination to favor PSI photochemistry (ΔAmax; Klughammer and Schreiber, 1994; Zygadlo et al., 2005; Klughammer and Schreiber, 2008). The light dependence of the P_700_ oxidation ratio (ΔA/ΔAmax; Klughammer and Schreiber, 1994; Zygadlo et al., 2005; DalCorso et al., 2008; Klughammer and Schreiber, 2008) was examined in *SV* and *SA* plants. The Far-red light (FR) intensity used was 102 μmol m^−2^ s^−1^ and a 100-ms saturating pulse (SP) of PPFD of 8,000 μmol m^−2^ s^−1^ was applied under background AL and FR.

### Conductance of the Electron Transport Chain (*g*_ETC_)

To estimate the conductance of the electron transfer chain (*g*_ETC_), we used a similar experiment setting to the previous section monitoring the redox state of PSI with slight modifications. The saturating pulse was given under darkness simultaneously with the termination of AL. Notably, a 100-ms width SP at a PPFD of 8,000 μmol m^−2^ s^−1^ was applied and the decay in absorbance followed upon transition from the 100 ms SP to darkness (Klughammer and Schreiber, 1994; Klughammer and Schreiber, 2008). This intensity was found to be saturating regardless the condition used. Accordingly, the application of a flash induced rapid rise in the absorbance (A) signal, with no decrease during the flash regime (100-ms, not shown). The absorbance decay curve under such conditions (ctrl or salt) approximated closely to a first-order kinetic and fitted well with a mono-exponential decay, yielding a rate constant. This is considered as the measure of the electron transfer chain conductance (Golding and Johnson, 2003; Stepien and Johnson, 2009).

### *In Situ* Histochemical Localization of Reactive Oxygen Species (ROS)

To detect the reactive oxygen species (ROS), a histochemical staining of the samples with nitroblue tetrazolium (NBT) was performed following Zhang et al. (2010) with minor modifications. Detached leaves were first vacuum-infiltrated in their appropriate solution (with or without NBT). For superoxide free radical (O^−^_2_ **^.^**) characterization, leaves were soaked in 6 mM NBT solution containing 50 mM sodium phosphate (pH 7.5) for 12 h under darkness. To detect hydrogen peroxide (H_2_O_2_), the detached leaves were immersed in 5 mM of 3, 3’-diaminobenzidine (DAB) solution containing 10 mM MES (pH 3.8) for 12 h under darkness. After that, the adaxial surface of the leaf was subjected to moderately high light (500 μmol m^−2^ s^−1^) for 1 h. The dark-blue spots reveal the interaction between NBT and the generated O^−^_2_^.^; however, the brown spots on the leaf reflect the interaction between DAB and formed hydrogen peroxide (H_2_O_2_) at the presence of peroxidase. Both reactions (DAB and NBT) were blocked through soaking the leaves in lacto-glycerol-ethanol (1:1:4 by vol). Chl was removed from the leaves before imaging by boiling leaves in their respective solutions (NBT or DAB) for 2 min then the solutions were discarded and leaves were re-boiled in water for two to three times (1 min each). Then leaves were incubated in alcohol (99.5%) as described by Zulfugarov et al. (2014) till complete removal of Chl. Afterwards, leaves devoid of Chl were preserved in 50% ethanol till photographed.

### RNA Extraction, Purification and qRT-PCR Analysis

Eight candidate housekeeping genes (Kumar et al., 2013) were screened to select an appropriate reference gene for *SA* and *SV.* These eight genes have been reported on *Setaria italica* (Foxtail Millet), representing different functional classes and gene families (Kumar et al., 2013). These genes are: viz., 18S rRNA (18S), elongation factor-1a (EF-1a), actin2 (Act2), alpha tubulin (Tub α), beta tubulin (Tub β), translation factor (TLF), RNA polymerase II (RNA POL II), adenine phosphoribosyl transferase (APRT; Kumar et al., 2013). In a recent study on *SA*, tubulin was used as a housekeeping gene (Karan and Subudhi, 2012a). Based on the similarity index between the sequences of each housekeeping gene in *SV* and *SA*, we obtained the highest similarity index in Tubulin alpha (Tub α), which is around 85%. In this study, we therefore selected Tub α as reference gene for qRT-PCR.

Total RNA was extracted from mature leaves using Purelink RNA Mini Kit (Invitrogen, Carlsbad, CA, USA) according to manufacturer’s instructions. Concentration of each RNA sample was measured using NanoDrop 2000 spectrophotometer (NanoDrop Technologies). Leaves were sampled from both species and total RNA was extracted using TRIzol Plus RNA Purification kit (Invitrogen Life Technologies, http://www.invitrogen.com). One microgram (1 μg) of total RNA was used to synthesize first strand cDNA with SuperScript VILO cDNA Synthesis Kit (Invitrogen Life Technologies, http://www.invitrogen.com). Quantitative real-time PCR (qRT-PCR) was performed using SYBR Green PCR Master Mix (Applied Biosystems, USA) with the fist strand cDNA as a template on a Real-Time PCR System (ABI StepOnePlus, Applied Biosystems lco., USA), with the following cycling parameters: 95°C for 10 s, 55°C for 20 s, and 72°C for 20 s. Primers for qRT-PCR were designed using Primer-Blast of the National Center for Biotechnology Information website (NCBI; https://www.ncbi.nlm.nih.gov) and Oligo 7 software. The primers for PTOX and Tubulin-alpha used for qPCR analysis were listed in **Table S2**. Relative expression of gene against housekeeping gene tubulin-alpha was calculated as: 2^−ΔΔCT^ (ΔCT = CT, gene of interest-CT, Tubulin-alpha), as described by Livak and Schmittgen (2001). Six complete biological and technical replicates were determined for the analysis.

### Detection of PTOX Contribution in Electron Transport in SA

To determine the contribution of the PTOX to the entire photosystem II (PSII) electron transfer, the leaves of either untreated (control) or salt-treated *SV* and *SA* were vacuum infiltrated with either water or with 5 mM *n*-propyl gallate (n-PG, 3,4,5-trihydroxy-benzoic acid-n-propyl ester; Sigma) or 50 μM DBMIB (2,5-dibromo-3-methyl-6-isopropyl-p-benzoquinone, Sigma). The stock solutions of *n*-PG were freshly prepared in ethanol and DBMIB in methanol.

To determine whether PTOX may play a role in electron transfer from PSII to O_2_, measurements of ETR_II_ were performed on leaves obtained from control and salt-treated *SA* and *SV* which were vacuum infiltrated with either water or a solution of the PTOX inhibitor *n*-propyl gallate (*n*-PG; 3,4,5-trihydroxy-benzoic acid-*n*-propyl ester; Joët et al., 2002; Josse et al., 2003; Kuntz, 2004; Rosso et al., 2006; Houille-Vernes et al., 2011; Sun and Wen, 2011; Trouillard et al., 2012; Shirao et al., 2013; Nawrocki et al., 2015).

### Western-Blot Analysis

For immunoblot (western-blot) analysis, thylakoids membranes were isolated according to the protocol of Cerovic and Plesnicar (1984). Thylakoids proteins were extracted from thylakoid membranes using 125 mM Tris–HCl buffer, pH 6.8, 20% glycerol, 4% (w/v) SDS, 5% (v/v) β-mercaptoethanol, 0.1% (w/v) bromophenol blue. Protein concentration was determined by the Bio-Rad protein assay kit (Bio-Rad Laboratories). The protein of the electrophoresis gel was transferred to nitrocellulose membrane as documented in Mudd et al. (2008). The specific antibodies raised against PTOX for both species (*SA* and *SV*) were designed by the company according the sequence homology between species which was 63% (see blast sequences alignment results in **Supplemental Data**).

For protein expression analysis, leaves from control and salt-treated plants were collected 12 d after initiating salt treatment. We used SDS-PAGE, to separate 29 μg protein from the thylakoid membrane samples. The protein on the electrophoresis gel was then transferred to nitrocellulose membrane and used for immunodetection. Western-blot band size was quantified by TanonImage technology software. For gene expression level, leaves from control and salt-treated plants for 12 d were collected and immediately frozen into liquid nitrogen. Then samples were used for RNA isolation and purification using Invitrogen PureLink RNA Mini Kit.

### Statistical Analysis

Statistical analysis was performed through one-way *ANOVA* using Tukey’s test. The difference between control and salt-treated samples for *SA* and *SV* was analyzed. Differences in the physiological parameters including ions (Na^+^ and K^+^) and total chlorophyll contents, the biophysical parameters encompassing Φ_PSII_, NPQ, ETRI, ETRII, *g*_ETC_, P_700_ oxidation ratio and eventually the PTOX expression level and its (PTOX) protein band size were all tested. The difference was labeled as being either strongly significant (***, *p ≤*0.001), or very significant (**, *p ≤*0.01), or significant (*, *p ≤*0.05), or not significant (, *p* ≥0.05).

## Results

### Chlorophyll *a* Fluorescence Induction and JIP-Test

**Figure 2:**
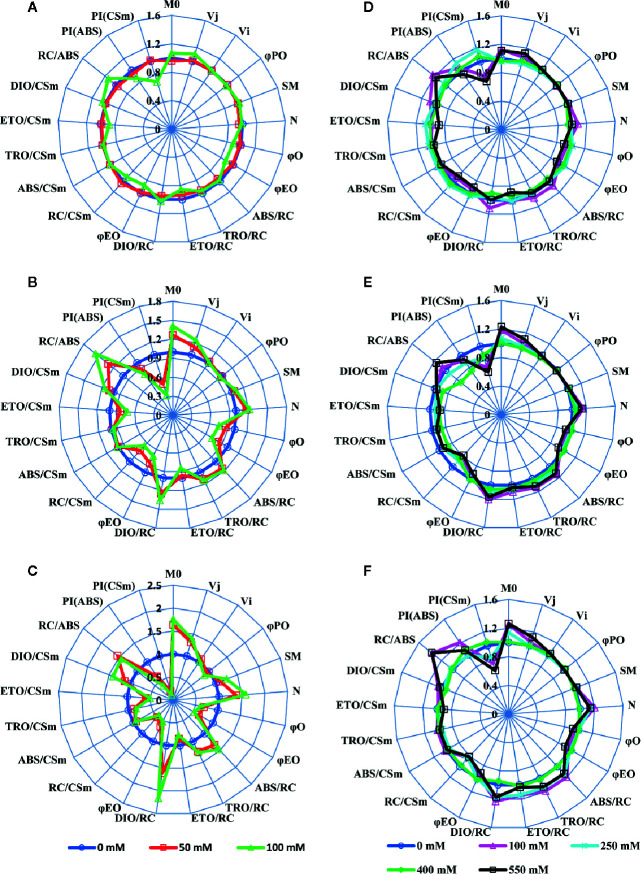
A ‘spider plot’ of selected parameters derived from the chlorophyll fluorescence OJIP curves for *SV* (left column, **A–C**) and *SA* (right column, **D–F**) treated with 0, 50 and 100 mM **(A–C)** and 0, 100, 250, 400 and 550 mM **(D–F)** NaCl for 12 days. All data of JIP test parameters were normalized to the reference 0 mM NaCl and each variable at the reference was standardized by giving a numerical value of the unit (1).

We used a JIP-test (Strasser et al., 2004) to unravel the salt stress impact on most of PSII parameters in both *SV* and *SA*. Results depicted in **Figure 2** were derived from the fast phase Chl *a* fluorescence induction curve, i.e. the OJIP curve. Salt stress treatment experiments show that, for *SV*, even moderate NaCl concentration (50 mM) increased the Fo level (data not shown) and the J-test of OJIP induction curves (data not shown). However, *SA* showed no/or slight difference in the OJIP induction curves for moderate (250 mM) and high (550 mM) NaCl concentrations compared to the control without NaCl. Therefore, the function of PSII was not affected for *SA*; however, it was strongly inhibited and/or damaged for *SV* even at relatively low NaCl concentration (50 mM). To study in detail the effects of NaCl on PSII in these two species, we evaluated the PSII parameters using the JIP-test (**Figure 1**, **Table S1**; Strasser et al., 2004). The JIP-test was evaluated from *SV* (A, B and C) and *SA* (D, E and F) exposed for 5 (A, D), 10 (B, E) and 15 d (C, F) to different NaCl concentrations. Herein, we observe that after 5 d of exposure to 100 mM NaCl, PSII parameters showed apparent change in *SV* (**Figure 2A**, green spider). The salt effect became more pronounced after 10 d of exposure to salt at either 50 or 100 mM (**Figure 2B**, red and green spiders). However, for *SA*, the deviation in the PSII parameters calculated with JIP-test was much lower and observable only for high NaCl concentration (550 mM) after 10 and 15 d’ exposure (**Figures 2E, F**, black spider). Therefore, PSII of *SV* was more sensitive to salt stress, as compared to *SA*.

### Sodium and Potassium Sequestration in Leaf Following NaCl Treatments

**Figure 3:**
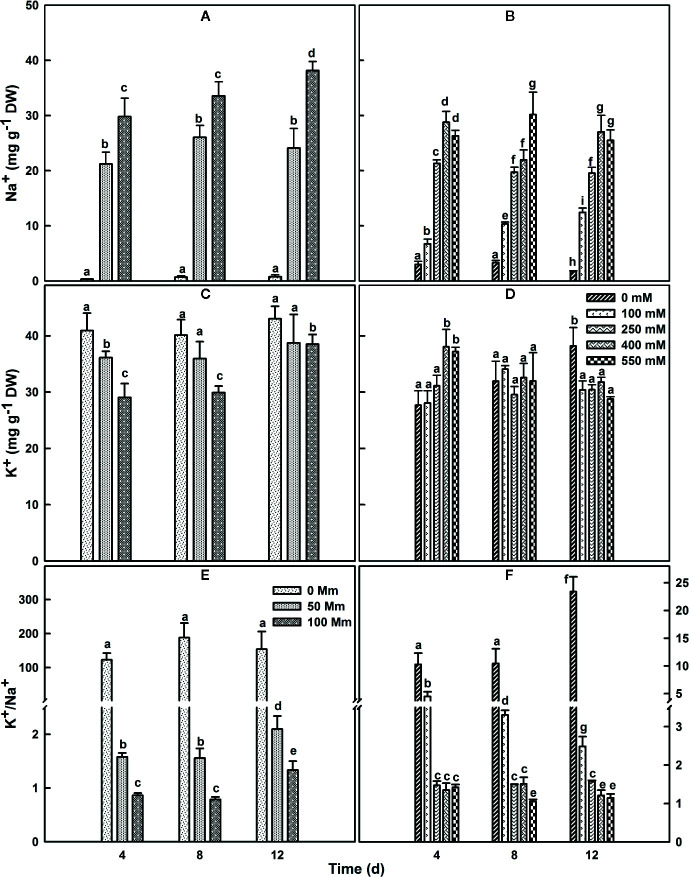
Changes in leaf Na^+^ **(A, B)**, K^+^ **(C, D)** contents and K^+^/Na^+^ ratio **(E, F)** over time in *SV* **(A, C, E)** and *SA* **(B, D, F)**. One-month old *SV* and 2 month-old *SA* were exposed to salt for up to 2 weeks. Plants were subjected to: 0, 50, and 100 mM NaCl for *SV* and 0, 100, 250, 400, and 550 mM NaCl for *SA*. Data represent the means of four to five replicates ± SE. The different letters above the bars indicate significant differences at *P* ≤0.05 among the treatments for the same species.

*SV* and *SA* plants were grown for 4 or 8 weeks before their exposure to different salt concentrations. Exposure of *SV* to NaCl levels higher than 100 mM resulted in plant destruction before finishing the experiment; so higher NaCl concentration treatments were not used or considered for *SV* in our current study. Subjection of *SA* to NaCl concentration till 550 mM did not result in a considerable lethality (mortality) for the plants. The concentration of Na^+^ in salt-untreated (control) leaf tissue was substantially higher in *SA* compared to *SV* (**Figures 3A, B**). This difference vanished after salt stress treatment, due to a quick accumulation of Na^+^ in the leaf of *SV*. The accumulation of Na^+^ in *SA* leaves was much lower at exogenous NaCl levels between 0 and 100 mM. Na^+^ accumulation enhanced enormously in *SV* leaves throughout the experiment (**Figure 3A**), while leaf Na^+^ level in *SA* increased less and gradually, even at higher external NaCl concentrations (**Figure 3B**). The Na^+^ levels estimated after 12 d NaCl treatment in *SA* exposed to 400 and 550 mM NaCl was nearly similar to that of *SV* subjected to only 50 mM NaCl (**Figures 3A, B**). At 100 mM NaCl, *SV* accumulated more NaCl in the leaf than *SA* under all salt concentration range (100–550 mM). This is owing to the exclusion of NaCl to the leaf surface for *SA*. This exclusion mechanism represents a second barrier of *SA* defense against high NaCl concentrations besides the sequestration of salt in the vacuole. Earlier study performed on halophyte *Aeluropus littoralis*, a species that can tolerate up to 800 mM NaCl, showed that an increase in leaf epidermis layer thickness was mainly due to cells swelling following salt sequestration in the leaf (Barhoumi et al., 2007).

*SA* and *SV* differed also in their K^+^ concentrations in the leaf. Herein, the concentration of K^+^ in leaf tissue of plants watered with medium without salt was lower by about 30% in *SA* leaves (**Figures 3C, D**). Following salt treatment, the K^+^ content of the leaf in *SV* decreased considerably, especially after 4 and 8 d treatment at 100 mM NaCl. However, in *SA*, there was an initial increase in K^+^ with the increase in NaCl concentration; after 12 d of treatments, the K^+^ concentration in the leaf gradually declined with an increase in the NaCl concentration (**Figure 3D**). This also reflected by the ratio k^+^/Na^+^ (**Figures 3E, F**), where we observed a dramatic decline in this ratio for *SV* but very less and mostly maintain stable with time course in *SA*, especially at NaCl concentrations higher than 250 mM (**Figures 3E, F**).

### Chl Content in Leaf and Non-Photochemical Quenching Decay Components: NPQ_fast,_ _slow_

**Figure 4:**
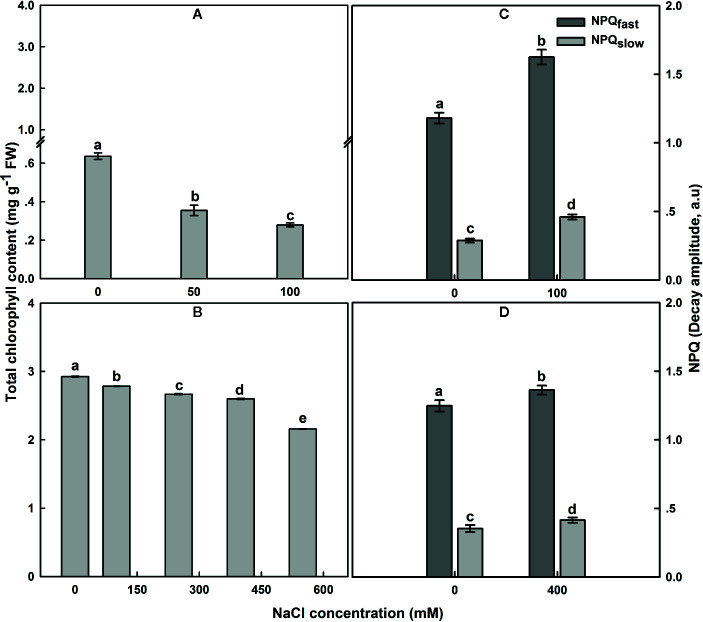
The effect of salt on the leaf total chlorophyll content in *SV* **(A)** and *SA* **(B)**. One month-old *SV* and two month-old *SA* were exposed to different NaCl levels as described in **Figure 2**. Leaves were collected 12 days after initiating salt treatment to determine chlorophyll concentration. For chlorophyll each data bar represents the mean of at least 10 replicates ± SE. Fast- and slow-relaxing components of NPQ (NPQ_f_ and NPQs) in leaves of *SV* **(C)** and *SA* **(D)** exposed to 0 and 100 **(C)** or 0 and 400 mM NaCl **(D)**. Measurements were carried out 16 days after initiating salt treatment at 25°C in the presence of 390 μl L^−1^ CO_2_. Leaves were illuminated with 800 μmol m^−2^ s^−1^ AL. Each data bar represents the means of at least six replicates ± SE. The different letters above the bars indicate significant differences at *P* ≤0.05 among the treatments for the same species.

The total Chl content in untreated *SA* leaves under salt treatment was around 4.5 times higher than that in untreated *SV* leaves (**Figures 4A, B**). Subjection of *SV* plants to NaCl entrained a gradual decrease in Chl content (**Figure 4A**); the total Chl concentration after 12 d of salt treatment with 50 and 100 NaCl declined by 42 and 58%, respectively. In contrast, treatment of *SA* with 50 and 100 NaCl did not result in a dramatic decline in the total Chl content except at NaCl concentrations higher than 250 Mm, e.g., at 550 mM NaCl treatment, there as a ~20% decrease in total Chl content (**Figure 4B**).

In *SV*, NaCl treatment resulted in an increase of NPQ, while NPQ remained similar or only slightly increased in *SA* at all NaCl concentrations (**Figures 4C, D**). The NPQ increase in *SV* might be resulted from a modulation of either a protective high-energy-state quenching or photoinhibition, which differ in the relaxation kinetics after AL illumination (Maxwell and Johnson, 2000). Measurements of NPQ were taken after 16 d exposure to 100 and 400 mM NaCl treatments for *SV* and *SA*, respectively (**Figures 4C, D**). The NPQ recovery under dark was measured to quantify the magnitude of each phase of NPQ dark decay. In *SV*, quantification of the relaxing phases of NPQ quenching (fast and slow) showed that most of quenching relaxed rapidly in the dark (NPQ_f_), indicating that it was high-energy-state quenching (**Figures 4C, D**). However, a part of the quenching was more conservative (NPQ_s_), revealing the occurrence of photoinhibition phenomenon in *SV* plants because of high salt stress treatment. Both components of NPQ quenching enhanced following salt stress treatment (**Figures 4C, D**). The increase in the total NPQ in *SA* was comparatively less and globally attributed to an increase in NPQ_f_ (photoprotection process).

### Electrons Flow to Molecular Oxygen Under Salt in Both C_4_ Species

**Figure 5:**
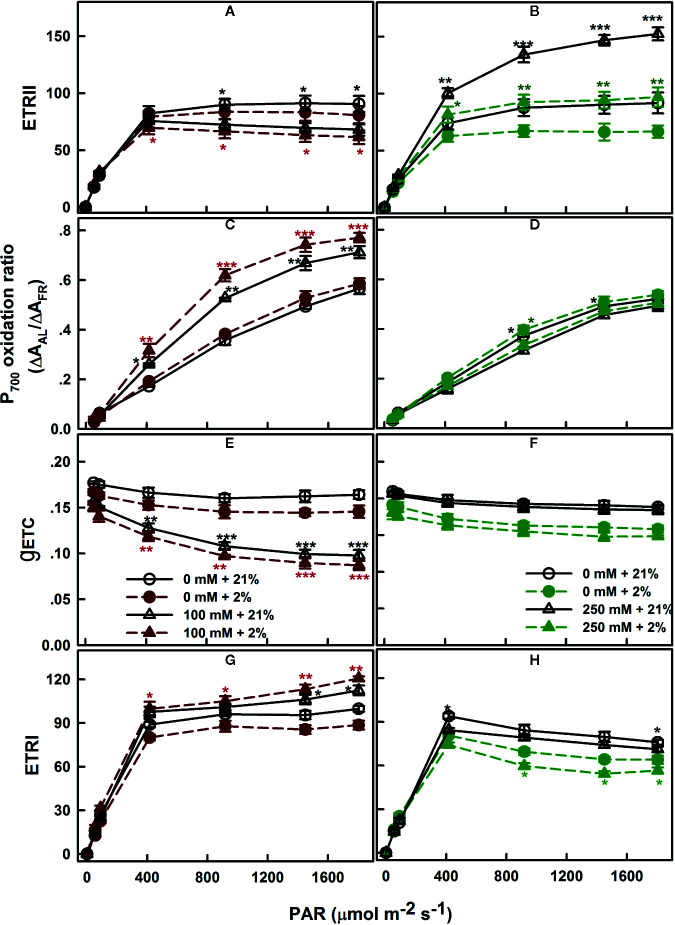
Oxygen dependence of electron transport: ETR_II_ **(A, B)**, P_700_ oxidation ratio **(C, D)**, *g*_ETC_ **(E, F)**, and ETR_I_ **(G, H)**, measured in leaves of *SV* (left column, **A**, **C**, **E**, **G**) and *SA* (right column, **B**, **D**, **F**, **H**) endured NaCl (triangles): 100 mM for *SV* and 250 mM for *SA*. Control plants (circles) were maintained in a NaCl-free medium. The measurements were performed, 12 days after initiating salt treatment, under saturating CO_2_ (2,000 μl L^−1^), at 25°C and in the presence of 21% (open symbols) or 2% (closed symbols) oxygen. Each data point represents the means of at least six replicates ± SE. The stars above the curves display the significance levels between control and salt treatment at *P* ≤0.05 (*), *P* ≤0.01 (**) or *P* ≤0.001 (***).

The electron generated by H_2_O splitting can be used by alternative sinks, in addition to the common sink to support NADPH generation. The most commonly known sinks are the reactions involving oxygen, including photorespiration and Mehler reaction (Chen et al., 2004; Shirao et al., 2013). To assess the relevance of these pathways, the electron flow dependent on the oxygen level was performed. *SV* and *SA* were subjected to different AL at a range of irradiance levels (AL, 0 to 1,806 μmol m^−2^ s^−1^) at the presence of saturating CO_2_ (2,000 μl L^−1^) and either 21 or 2% of O_2_. Regardless the degree of NaCl treatments, the ERT_II_ in both species reached its maximum at around 400 μmol m^−2^ s^−1^ (**Figures 5A, B**). Exposure of control leaves during measurement to low oxygen level (2%) caused a decrease in ETR_II_ at saturating irradiances.

Measurements of the redox state of P_700_, the primary electron donor of PSI, revealed slight effects of the various oxygen percentage levels (2 and 21%) in salt-untreated (control) plants. With increasing irradiance, P_700_ gradually becomes more oxidized in both species *SV* and *SA* (**Figures 5C, D**). Despite the proportion of oxidized P_700_ (P_700_^+^) was insensitive to the oxygen concentration in salt-untreated plants, the conductance of the ETC (*g*_ETC_; **Figures 5E, F**) declined and the ETRI follows the same trend and decreased by the same amount (**Figures 5G, H**).

*SV* exposed to 100 mM NaCl showed lower ETR_II_ at high CO_2_ than the control plants (**Figure 5A**). As in the control, electron transport through PSII decreased slightly under low O_2_ (**Figure 5A**). Conversely, exposure to NaCl caused an increase in the ETR_II_ in *SA* compared to untreated plants; this increase in electron transfer through PSII was completely revoked under low O_2_ concentration (**Figure 5B**).

The fraction of oxidized P_700_ (P_700_^+^) in salt-treated *SV* was significantly higher under low O_2_ (p ≤0.05; **Figure 5C**). This is accompanied by to a negligible decline in the *g*_ETC_, resulted in a slight increase in the electron flow through PSI potentially *via* cyclization across FRQ (**Figure 5G**). Conversely, in *SA*, PSI ETR (ETR_I_) in the absence or presence of salt (250 mM) decreased at low O_2_ (**Figure 5H**). This caused by an enhancement in P_700_ oxidation and a fall in *g*_ETC_ (**Figures 5D–F**).

### Activity of NAD(P)H Dehydrogenase (NDH)-Dependent Cyclic Electron Flow in Both Species Under Salt Stress

**Figure 6:**
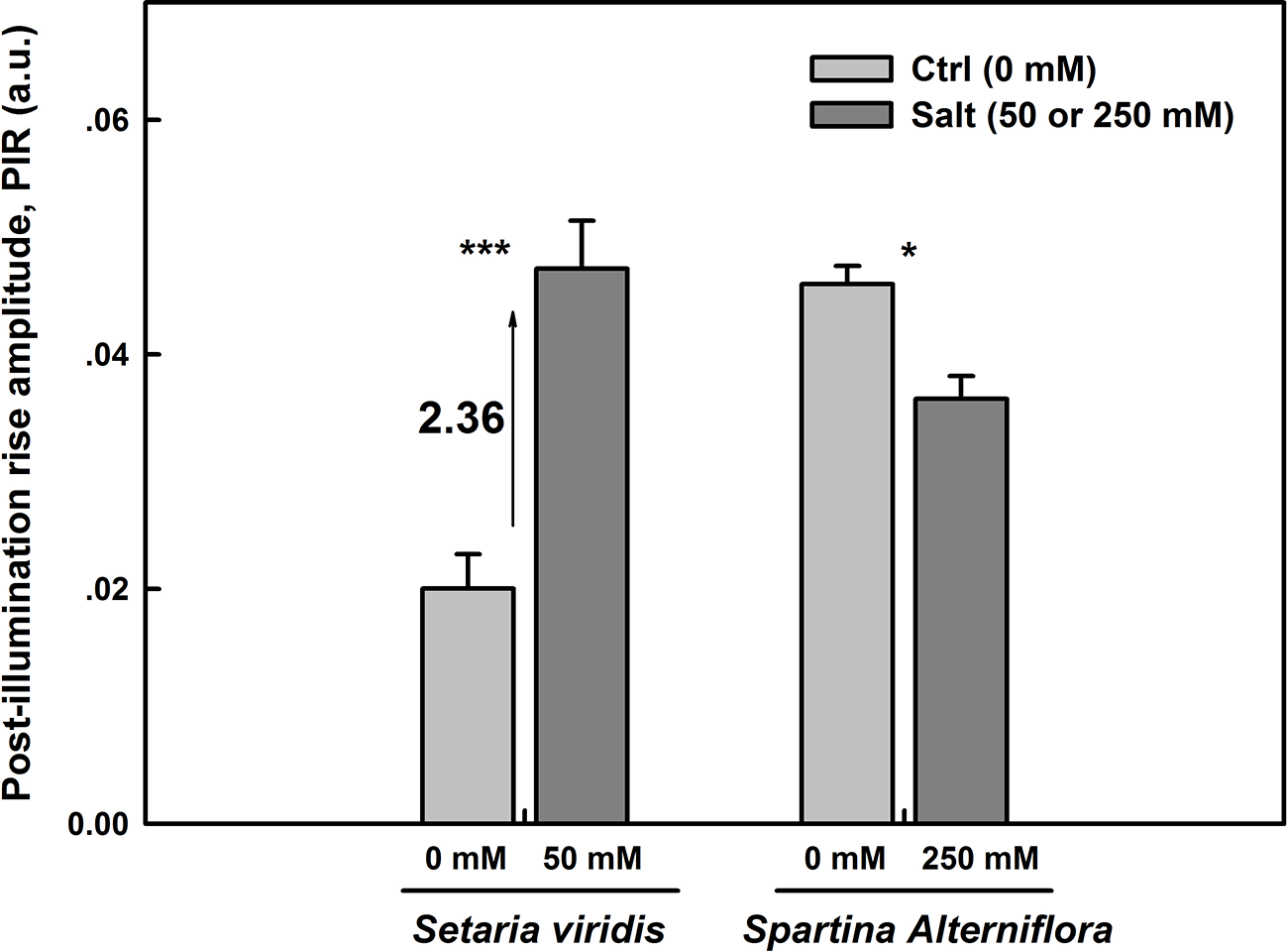
NDH-dependent CEF pathway assessed as the post-illumination F_o_ rise in plants grown on either salt-free medium (ctrl) or subjected to 50 or 250 mM NaCl for *SV* and *SA*, respectively, during 12 days. The post-illumination F_o_ rise was recorded in the dark after switching off 5 min illumination with AL (325 μmol m^−2^ s^−1^). Each data bar represents the means of at least 10 replicates taken on different leaves ± SE. The stars above the bars display the significance levels between control and salt treatment at *P* ≤0.05 (*) or *P* ≤0.001 (***).

NDH cyclic pathway activity around PSI was assessed as the post-illumination rise (PIR) of F_o_ Chl fluorescence was monitored after switching off AL (Essemine et al., 2016). The magnitudes of PIR for *SV* and *SA* under both control and salt stress conditions were displayed in **Figure 6**. Under normal conditions, we observe more than two times higher NDH activity in *SA* than in *SV* (**Figure 6**). The results show as well an increase in the NDH in leaves of *SV* plants endured 50 mM NaCl for 12 d by about 2.36 times (**Figure 6**). However, *SA* plants exposed for the same time period (12 d) to 250 mM NaCl exhibited a significant decrease by about 25% (p <0.05) in the NDH activity (**Figure 6**) compared with the control. This is very likely attributable to the activation of PTOX in *SA* under salt stress. Hence, the activity of PIR declines in *SA* in favor of that of PTOX. This reflects the existence of an efficient competition between these two pathways (PTOX and NDH) for the oxidation/reduction of the PQ pool, respectively. Eventually, the oxidation of the PQ pool by PTOX overcomes its re-reduction by NDH cyclic (**Figures 6** and **8**). So far, PTOX may represent an alternative pathway to cyclic and linear routes for the protection of *SA* against intersystem over-reduction and minimize or avoid damages to both photosystems (PSI and PSII). Thereby, it may function as a safety valve for the photosynthetic transport chain. In this regard, our findings are in line with that of Ahmad et al. (2012), where authors have shown a decrease in the PIR in tobacco overexpressing PTOX from *Chlamydomonas reinhardtii* (Cr-PTOX) compared to wild type, WT (Ahmad et al., 2012) and they demonstrated that the decrease in PIR is attributed to the enhanced activity of PQ pool oxidation by the high level of PTOX protein in the over-expressed line.

### Plastid Terminal Oxidase (PTOX) as a Plastohydroquinone : Oxygenoxidoreductase

The improved efficiency and/or the additional turnover of PSII under salt treatment in *SA* at the presence of 21% oxygen, compared to either control with 21% O_2_ or 250 mM NaCl with 2% O_2_, is very likely attributed to electron transfer directly to molecular oxygen (O_2_). Since experiments were conducted under a saturating CO_2_ concentration of 2,000 μl L^−1^, we exclude the contribution of photorespiration to this effect. Usually, the photo-reduction of O_2_ may happen at the PSI acceptor side *via* the Mehler reaction; nevertheless, the lack of sensitivity of PSI parameters to oxygen suggests that this is unlikely the reason, or at least not the only reason. So here we test the possibility that the putative quinone-oxygen oxidoreductase, the plastid terminal oxidase (PTOX) or IMMUTANS protein (Shahbazi et al., 2007; Heyno et al., 2009) might have played a role as well for *SA*.

**Figure 7:**
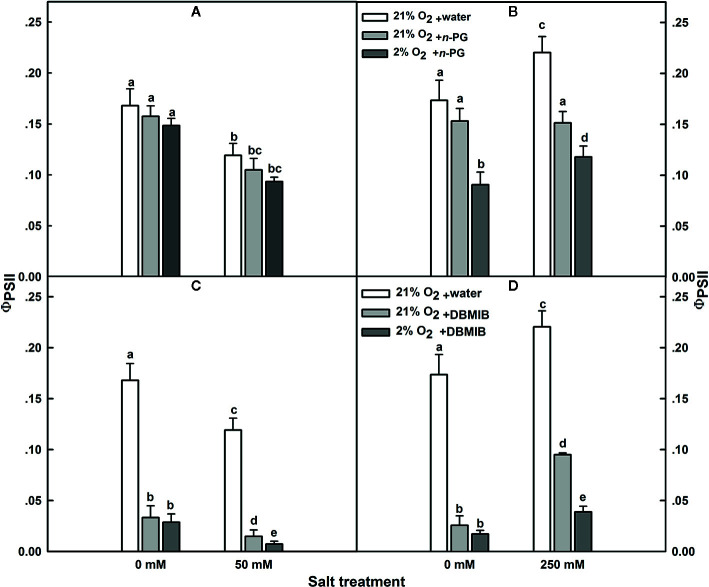
Effects of *n*-PG and DBMIB on PSII photochemical efficiency Φ_PSII_ measured in leaves of either *SV* **(A, C)** or *SA* **(B, D)** detached from plants subjected to: 0 and 50 (for *SV*); or 0 and 250 mM NaCl (for *SA*). Measurements were carried out 12 days after initiating salt treatment at 25°C in the presence of 390 μl L^−1^ CO_2_. Leaves were illuminated with 800 μmol m^−2^ s^−1^ red AL. Leaves were vacuum infiltrated with water (white bars) or with 5 mM *n*-PG **(A, B)** or 50 μM DBMIB **(C, D)** in the presence of 21% (gray bars) and 2% (black bars) oxygen. Each data point exhibits the means of at least six replicates ± SE. The different letters above the bars indicate significant differences at *P* ≤0.05 among the treatments for the same species.

In *SV*, PSII quantum yield (ɸ_PSII_) was insensitive to *n*-PG, regardless whether the plants have been exposed to salt stress or not (**Figure 7A**). The same was observed for salt-untreated (control) *SA*. However, in *SA* subjected to 250 mM NaCl, ɸ_PSII_ was obviously sensitive to *n*-PG (**Figure 7B**). ɸ_PSII_ measured 12 d after initiating NaCl treatment was reduced by about 32 and 45%, in leaves infiltrated with 5 mM *n*-PG, in the presence of 21 and 2% O_2_, respectively (**Figure 7B**), falling thereby to the control level or even slightly lower (**Figure 7B**). Interestingly, at low O_2_ in salt-stressed plants, we observed a decrease in the ɸ_PSII_. This suggests strongly that molecular oxygen (O_2_) may act as a terminal electron acceptor by oxidizing the plastoquinol (PQH2).

The effect of *n*-PG suggests a potential activity of PTOX, which is situated on the stromal side of the membrane in *SA* though this does not exclude a potential contribution of the Mehler reaction to electron transport. To measure the electron flow to oxygen excluding any contribution of the Mehler reaction, leaves were infiltrated with the cytochrome b_6_/f (Cytb_6_/f) inhibitor dibromothymoquinone or 2,5-dibromo-3-methyl-6-isopropylbenzoquinone (DBMIB), a specific inhibitor of the Q_o_-binding site (Malkin, 1981; Malkin, 1982; Rich et al., 1991; Schoepp et al., 1999). In *SV*, this almost completely abolished the ɸ_PSII_ and thereby the electron flow beyond the cytb_6_/f, regardless the NaCl treatment (**Figure 7C**). In control *SA* leaves, DBMIB also strongly inhibited PSII, though a residual ɸ_PSII_ and also electron transfer remained. Regardless the O_2_ concentration used (2 or 21%), DBMIB dramatically declined ɸ_PSII_ (**Figure 7D**). In salt-stressed *SA* leaves, DBMIB only partially inhibited ɸ_PSII_; however, decreasing the O_2_ concentration (2%) resulted in a greater ɸ_PSII_ inhibition (**Figure 7D**). The extent of DBMIB-insensitive ɸ_PSII_ but sensitive to oxygen decrease (2%) was similar to that of *n*-PG-sensitive ɸ_PSII_ in the same leaves (**Figures 7B, D**).

The dramatic decline in ɸ_PSII_ in the presence of DBMIB at low O_2_ in salt treated *SA* leaves (**Figure 7D**) might be explained as a double restriction in the electrons flux beyond PSII. First limitation due to the blockage (or shortage) in the electrons flow towards PSI due to the presence of DBMIB and the second curtailment is tightly linked to the drop in the O_2_ level (2%).

**Figure 8:**
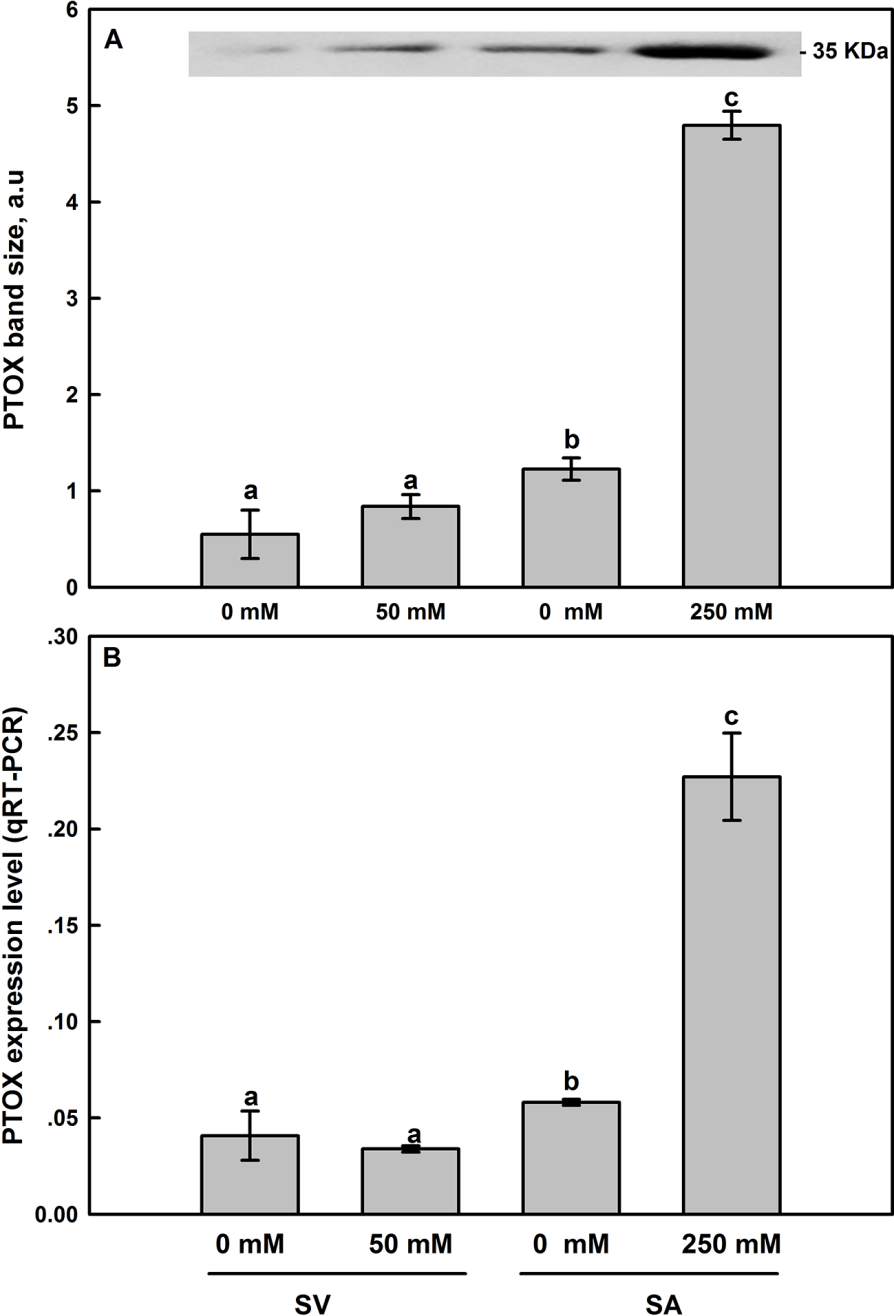
Effect of salt treatment on PTOX protein expression **(A)** and the PTOX gene expression level assessed by q-PCR analysis **(B)** in leaves of *SV* and *SA* subjected to 0 and 50 (for *SV*) or to 0 and 250 mM NaCl (for *SA*). For more details on the sampling method, samples preparation, gel running and bands analysis and quantification, please refer to *Materials and Methods* (*Western-Blot Analysis* section). The synthesized cDNA was used for the q-PCR analysis of PTOX. Data points represent the mean of around five replicates for western SDS-PAGE ± SE and six replicates for qRT-PCR. Insert of panel A shows typical bands from an original blot, loaded on an equal protein basis. The different letters above the bars indicate significant differences at *P* ≤0.05 among the treatments for the same species.

Western-blot analyses of thylakoid membrane extracts of *SA* and *SV* using antibodies raised against *Zea mays* PTOX revealed the presence of a 35-kDa band in both species (**Figure 8**). For untreated plants, *SA* showed higher protein abundance than in *SV*. In the latter (*SV*), salt treatments resulted in a slight increase in the PTOX abundance (**Figure 8A** and inset), though the expression level of PTOX transcript insignificantly decreased (**Figure 8B**). In *SA*, treatment with 250 mM NaCl elevated PTOX abundance by 3–4 times compared to the control (**Figure 8A** and inset). Similarly, the transcript abundance of PTOX was also elevated under NaCl treatment by the same amount (**Figures 8A, B**).

### Reactive Oxygen Species Generation Under Salt in C_4_ Species

**Figure 9:**
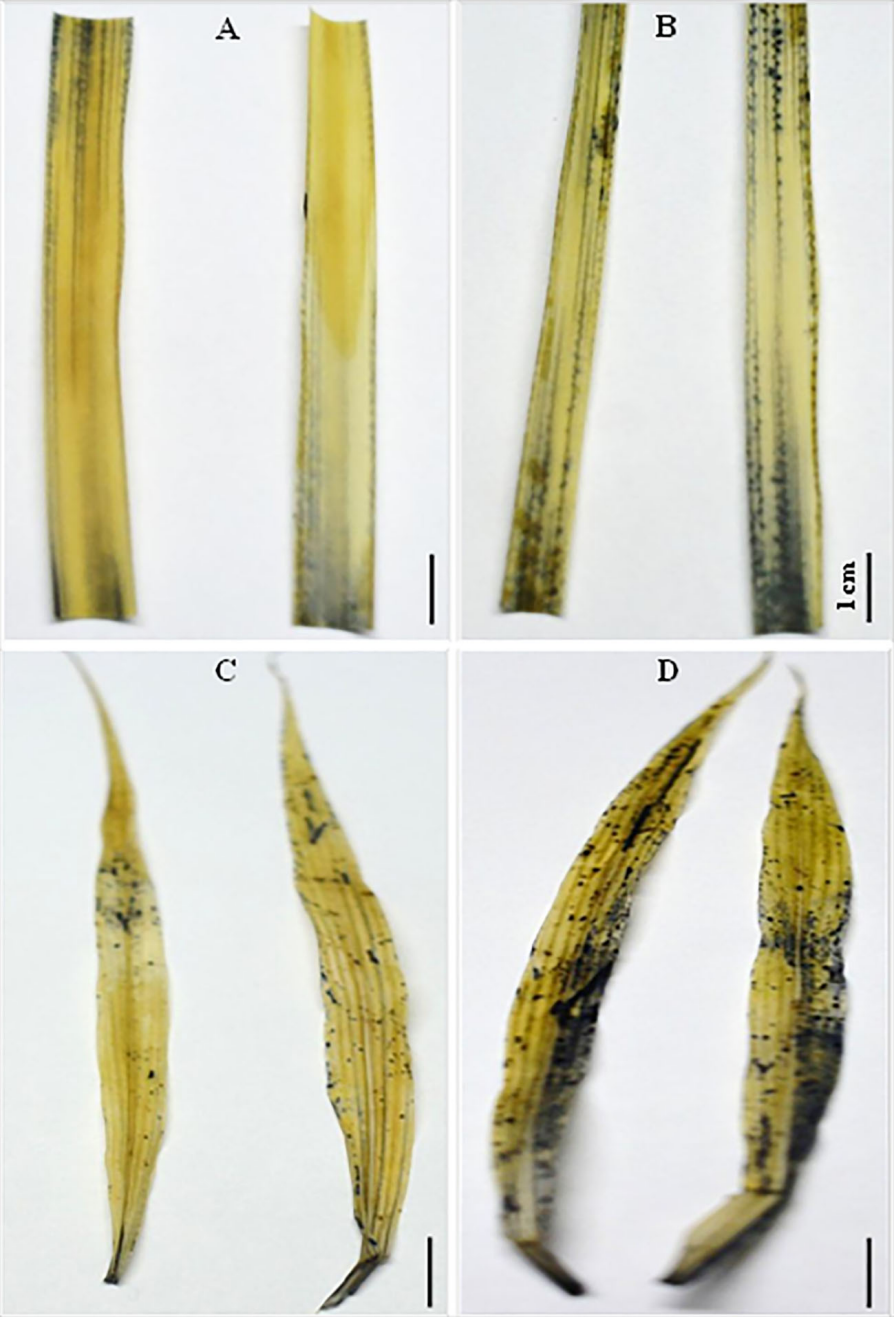
Histochemical staining of *SV* **(C, D)** and *SA* **(A, B)** leaves obtained from control untreated **(A, C)** or salt treated **(B, D)** for *SV* at 50 mM and *SA* at 250 mM during 12 days with 6 mM NBT (nitroblue tetrazolium). Dark-blue staining reveals the interaction of superoxide radical (O^−^_2_) with NBT (500 μmol m^−2^ s^−1^) in leaves following salt stress treatment. The white bar represents the scaling bar of 1 cm length.

**Figure 10:**
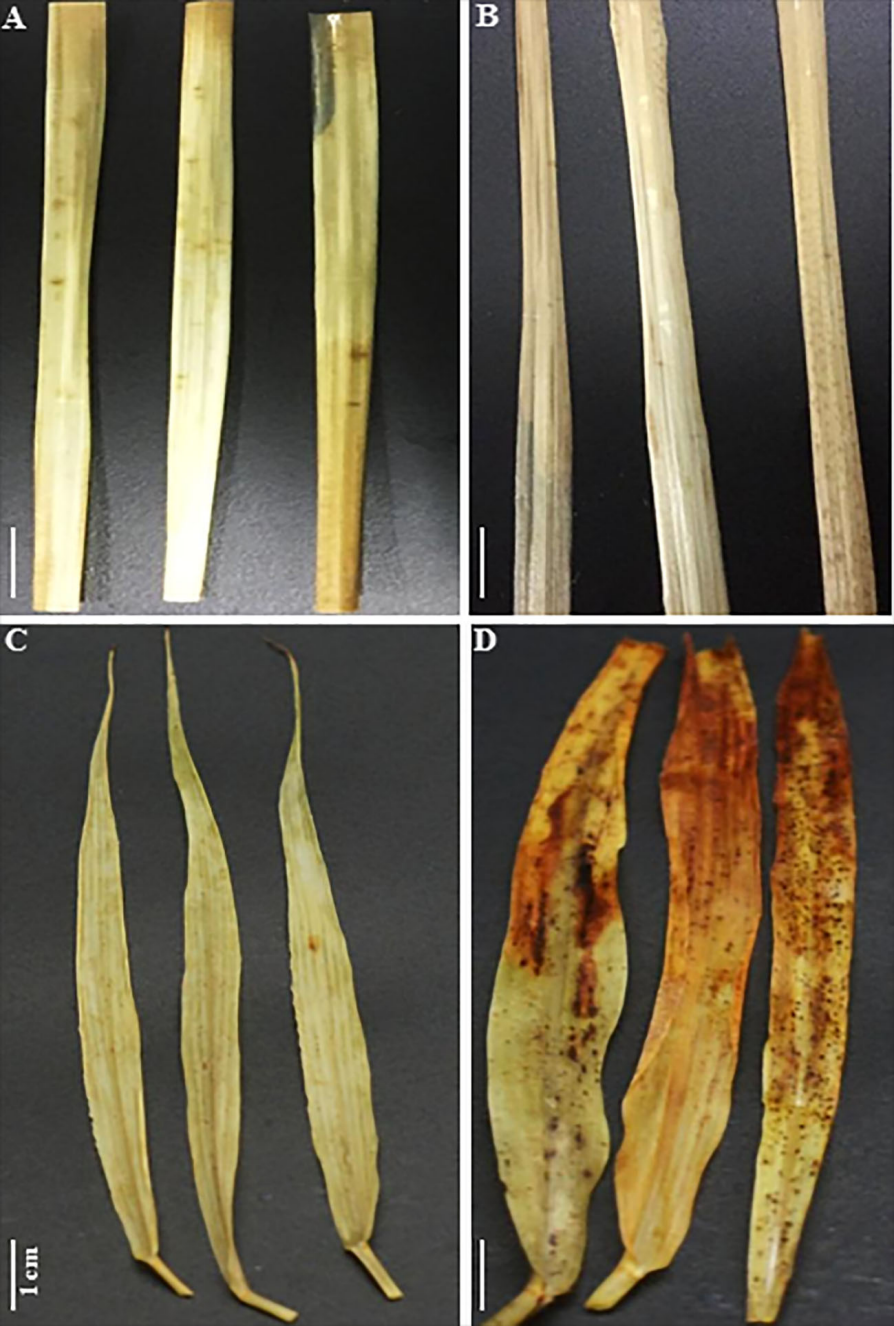
Histochemical staining of *SV* **(C, D)** and *SA* **(A, B)** leaves obtained from control untreated **(A, C)** or salt treated **(B, D)** for *SV* at 50 mM and *SA* at 250 mM during 12 days with 5 mM DAB (diaminobenzidine). Brown spots reflect the interaction of hydrogen peroxide (H_2_O_2_) with DAB under light (500 μmol m^−2^ s^−1^) in leaves following salt stress treatment. The white bar represents the scaling bar of 1 cm length.

Histochemical staining with nitroblue tetrazolium (NBT) shows the appearance of dark-blue spots on the edge of *SA* leaves exposed to 250 mM NaCl for 12 d (**Figure 9B**). This dark-blue staining reveals the interaction between NBT and the generated superoxide free radical (O^−^_2_ **^∙^**) following exposure to moderately high light (500 μmol m^−2^ s^−1^). However, these dark-blue spots were spread all over the surface of *SV* leaves subjected to 50 mM salt for 12 d (**Figure 9D**), suggesting that salt treatment dramatically increased the production of O^−^_2_ **^∙^** in *SV*. Similarly, histochemical staining using diaminobenzidine (DAB) showed no visible brown spots on either control or salt-treated *SA* leaves (**Figures 10A, B**). In contrast, *SV* treated with only 50 mM NaCl for 12 d shows a widespread presence of brown spots on the leaf surface (**Figures 10C, D**).

## Discussion

### Salt Stress Induced Up-Regulation of Electron Flow Through the PTOX Activity in *SA*

There is a huge difference between *SV* and *SA* regarding their physiological response to salt. Here, we found that in *SA*, with time, either a stable or an increase in the K^+^/Na^+^ was observed (**Figures 3E, F**). This maintenance or increase in the K^+^/Na^+^ is a major trait associated with salt tolerance (Shabala and Pottosin, 2014). Na^+^ tolerance is associated with SOS1 antiporter localized to the root epidermis (Shi et al., 2002). Mostly, halophytes exhibit higher SOS1 abundance (Oh et al., 2009). Therefore, exclusion of Na^+^ should also be a mechanism involved in salt-tolerance in *SA.* In addition to this known mechanism of salt tolerance, here our data suggest that under salt, *SA* gained increased salt tolerance through increased electron flow through PTOX.

Firstly, under normal growth conditions, i.e. when there was no salt stress, the NDH-dependent CEF activity was more than two times higher in *SA* than in *SV* (**Figure 6**). However, after NaCl treatment, the NDH activity was enhanced by 2.36 times in *SV* but decreased by about 25% in *SA*, compared to their respective control (**Figure 6**). After exposure to salt stress, the J-step of OJIP curves was significantly enhanced for *SV* compared to *SA* (data not data). The increase in the J-step constitutes an indicator of a more reduced PQ pool and a more exacerbated Q^-^_A_ (primary electron acceptor of PSII) accumulation under salt stress (Haldimann and Strasser, 1999). This leads to a strong PSII acceptor side limitation and a high PQ pool over-reduction in *SV* compared to *SA*. Furthermore, we found that under salt stress, the level of NPQ was similar between *SA* and *SV,* i.e. the incident light energy was not more dissipated in the form of heat in *SA*, as compared to *SV.* There must be a major source of electron which accept electron in *SA* under salt stress.

Second, experiments using inhibitors suggest that PTOX is a major sink of electrons in *SA* under salt. To test this, we examined the PSII photoinhibition following salt stress in presence of *n*-PG (PTOX inhibitor) or DBMIB (Q_o_-binding site of Cytb_6_f inhibitor) at atmospheric CO_2_ (390 μl L^−1^ CO_2_) and in presence of 2 or 21% O_2_ (**Figure 7**). Our results revealed that the restriction in electrons flow towards PTOX (*n-PG*) has little effect on the Φ_PSII_ in *SV* (**Figure 7A**) but significantly decreased Φ_PSII_ in *SA* under both conditions (normal and salt), especially in the presence of low O_2_ (**Figure 7B**). This reflects that a proportion of electrons from PSII is sensitive to both to *n*-PG and O_2_ (13%, **Figure 7B**). This provides an evidence that an efficiently operating PTOX in *SA* but not in *SV* under salt stress. In fact, even under non-salt condition, there is a proportion of electron from PSI flow into PTOX driven reactions.

Thirdly, using DBMIB, we observed that in *SA,* as compared to *SV*, under high NaCl treatment, the PSII was less photoinhibited, especially at the presence of 21% O_2_ (**Figures 7C, D**). This is possibly because under severe salt stress, electrons can be used to reduce O_2_ in *SA* through PTOX without passing through Cytb_6_f. Consistent with this possibility, we observed an enhancement in the primary PSII electron transfer rate under salt in the presence of 21% O_2_ and saturating CO_2_, 2,000 μl L^−1^ (**Figure 5B**). Under 2,000 ml·L^−1^ CO_2_, the electron flux towards photorespiration pathway is minimized, leading to a reduction and/or restriction in the photorespiration process as a major sink for the reducing power. This provides further evidence that PTOX may functions as a major electron sink in *SA* under salt stress. Furthermore, in line with this notation, this enhancement of electron transfer rate was not observed under low O_2_ (2%) under salt stress (**Figure 5A, B**). The gene expression and Western-blot analysis also showed that under salt stress, there were increased amount of PTOX RNA and protein abundance in *SA*, but not in *SV* (**Figure 8**). Therefore, upon salt stress, the *SA* shows drastically increased electron flow into TPOX. The increase of PTOX levels have also been reported earlier in plants under stress, e.g. exposure of tomato to high light (Shahbazi et al., 2007) or thellungiella to salt stress (Stepien and Johnson, 2009).

### PTOX as a Safety Valve in *SA* Under Salt Stress to Protect Photosystems From Over-Reduction

PTOX is an interfacial membrane protein (Berthold and Stenmark, 2003) attached to the stromal-side of the thylakoid membrane (Lennon et al., 2003). PTOX is involved in the carotenoid biosynthesis (Carol and Kuntz, 2001) and has been implicated in the oxidation of the plastoquinol pool, PQH_2_ (Joët et al., 2002). Similar to the increase of PTOX under salt conditions in *SA*, the PTOX levels have been found to increase in higher plants subjected to abiotic stress such as high temperatures, high light and drought (Quiles, 2006; Díaz et al., 2007; Ibáñez et al., 2010), low temperatures and high light (Ivanov et al., 2012), salinity (Stepien and Johnson, 2009) and in alpine plants at low temperature and high UV exposure (Streb et al., 2005; Laureau et al., 2013), implying a generic role of PTOX under stress.

Data from this study provide new evidence for the protective role of PTOX under salt stress. F_o_ of Chl *a* fluorescence (OJIP) was found to increase in *SV* but was not changed or changed little for *SA* (data not shown). After exposure to salt stress, the J-step of OJIP curves was significantly enhanced for *SV* compared to *SA* (data not shown). The increased J level is an indicator of an exacerbated PQ pool reduction and a pronounced Q^-^_A_ (primary electron acceptor of PSII) accumulation under salt (Haldimann and Strasser, 1999). This leads to a strong PSII acceptor side limitation and a high PQ pool over-reduction in *SV* compared to *SA*. In this regard, similar results have been reported by Shahbazi et al. (2007). These authors proved similar effect of high light treatment on the mutant of tomato *ghost* (*gh*) defective in PTOX compared to the control San Marzano (SM) (Shahbazi et al., 2007). The data from this study, together with these earlier studies, suggests that PTOX can oxidize over-reduced PQ pool and hence provides protective roles.

As a reflection of the protective role, *SA* plants grew normally at a moderate salt stress and even survived under NaCl concentrations up to 550 mM NaCl without significant mortality. The Chl content of leaves did not drop significantly, particularly at NaCl concentrations below 250 mM (**Figure 4B**) and both stomatal conductance (*gs*) and assimilation at atmospheric CO_2_ concentrations (A) were maintained (Essemine et al., unpublished data). By comparison, *SV* was unable to survive at NaCl level higher than 100 mM for two weeks; even at NaCl concentrations lower than 100 mM, the Chl content of *SV* dropped drastically by about 42 and 58% after 12 d exposure of *SV* to 50 and 100 mM NaCl, respectively (**Figure 4A**), concurrent with a dramatic decline in both *gs* and *A* (Essemine et al., unpublished data).

The protective role is clearly shown by changes in the linear electron transfer rates under NaCl treatments. In *SV*, under salt stress, we observed a decrease in linear electron transfer rate (LEF), as shown by the decrease in the *g*_ETC_ at saturating CO_2_, which has a concentration of 2,000 μl L^−1^ at either 21 or 2% O_2_ levels (**Figure 5**). Such decrease is common among C_3_ species under stress, e.g. drought (Golding and Johnson, 2003), salt (Stepien and Johnson, 2009), and anaerobiosis (Haldimann and Strasser, 1999). In *SA*, in contrast, there was no apparent decrease in LET under salt (**Figure 5B**); which suggests that the photosystem II in *SA* under stress was well protected. Consistent with these differential capacities to protect photosystem under salt, we observed much higher accumulation of ROS in *SV* compared to *SA*, even though the salt concentration used to treat *SV* was 50 mM, while that used to treat *SA* was 250 mM (**Figures 9** and **10**). The reactive oxygen species (ROS) detected here may include highly reactive singlet oxygen (Kearns, 1971), the superoxide anion radical (O^−^_2_ **^∙^**) and hydrogen peroxide, H_2_O_2_ (Fridovich, 1997). The severe damage of salt to photosystem in *SV* is also reflected by a swelling in the chloroplast structure for *SV* after exposure to salt (Essemine et al., unpublished data). Altogether, these data suggest that having higher PTOX activity under salt (**Figure 8**) may contributed to the protection of chloroplast structure and function, as shown by maintenance of the photosynthetic linear electron transfer, chlorophyll a content, and less accumulation of ROS in leaves.

It is worth mentioning here that the protective function of PTOX has been studied earlier through transgenic approaches. However, the data obtained so far from transgenic experiments are still not conclusive. When PTOX from *C. reinhardii* was transferred into tobacco (Ahmad et al., 2012), it resulted in growth retardation; furthermore, instead of inducing increased resistance to high light, it led to increased vulnerability to high light. The ortholog of PTOX in *Arabidopsis* has also been studied using both mutant and over-expression lines; which however, did not provide proof for a role of PTOX in the modulation of PQ redox status (Rosso et al., 2006). In tobacco, however, over-expression of PTOX led to increased photoprotection under low light but increased vulnerability under high light, or which the authors suggest that the PTOX can only provide a sufficient photoprotection when the reactive oxygen species generated by PTOX can be effectively detoxified (Heyno et al., 2009). However, the increased susceptibility of plant growth to high light was not shown in tobacco over-expressing PTOX from *Arabidopsis* (Joët et al., 2002). In high mountain species *Ranunculus glacialis*, the rate of the linear electron transfer far exceeds the rate of consumption of electrons for carbon assimilation rate under different temperature and light levels; especially under 21% O_2_ and high internal CO_2_ concentration (*C_i_*), suggesting a major role of PTOX in photoprotection (Streb et al., 2005).

### PTOX and NDH-Mediated Cyclic Electron Transfer

Under stress conditions, the cyclic electron transfer rate usually increases as demonstrated for spinach (Breyton et al., 2006) and *Arabidopsis* (Shikanai, 2007; Strand et al., 2015). In contrast, here we show that in *SA*, which has a great capacity of channeling electrons to PTOX, the rate of cyclic electron transfer rate decreased (**Figure 6**). This is clearly shown by data from the post-illumination Fo rise (PIR) signal, which was used here to assay NDH (Burrows et al., 1998). Using this method, we found a stark contrast in the responses of NDH-dependent CEF and PTOX to salt stress between species (*SA* and *SV*). In *SV*, the strong stimulation of NDH-dependent CEF following salt stress (236%) was concurrent to a nearly stable PTOX level (**Figures 6** and **8**). However, in *SA*, we observed a decline in the NDH-dependent CEF (**Figure 6**) together with an increase of PTOX expression levels, which was up-regulated by up to four times compared to the control as assessed by both RNA-expression abundance and protein abundance (**Figure 8**).

Our finding about the negative relationship between PTOX and NDH-CEF is in line with a number of earlier reports. In this regard, Ahmad et al. (2012) have reported a dramatic decline in the NDH activity in tobacco expressing PTOX from green algae (Cr-PTOX1). Furthermore, PTOX may efficiently compete with CEF for the plastoquinol (PQH_2_) in the CRTI-expressing (carotene desaturase) lines (Galzerano et al., 2014). Joët et al. (2002) also showed a decrease in the NDH-dependent CEF flux in tobacco transgenic lines expressing PTOX from *Arabidopsis*. The activity of cyclic electron transfer is regulated by an array of mechanisms, including redox status (Breyton et al., 2006; Takahashi et al., 2013), H_2_O_2_ (Strand et al., 2015), metabolite levels (Livingston et al., 2010), Ca signaling (Lascano et al., 2003; Terashima et al., 2012), and even phosphorylation of NDH components (Lascano et al., 2003). It is likely that NaCl induced differential changes in the NDH and PTOX, though mechanism is complexly unknown. It is possible that some internal signals from chloroplast, such as redox status of chloroplast electron transfer chain, or particular compound in the photosynthetic carbon metabolism, or even H_2_O_2_, might differentially regulate PTOX and NDH-CET. Mechanisms how PTOX and NDH-CET were differentially regulated under NaCl needs further elucidation. It is worth mentioning here that *SA* has been used as a model halophyte grass species to study adaptation to plants to salt stress and to mine salt stress-tolerance genes (Subudhi and Baisakh, 2011). Several earlier reports have demonstrated the utility importance of genes from this halophyte (*SA*) in the amelioration of crop salt resistance (Baisakh et al., 2012; Karan and Subudhi, 2012a; Karan and Subudhi, 2012b). Therefore, elucidation of how PTOX and NDH-CET respond under NaCl to protect photosystem and leaf functioning can help develop new strategy to protect photosystems under salt stress.

## Data Availability Statement

All datasets presented in this study are included in the article/**Supplementary Material**.

## Author Contributions

Conceptualization: JE, X-GZ. Methodology and design: JE, X-GZ, M-JL. Experiments: JE, M-JL, MQ, NK, SP, SS, GC. Investigation: JE, M-JL, MQ. Writing: JE, X-GZ. Funding Acquisition: X-GZ. Resources: JE, CG, X-GZ. Supervision: X-GZ. All authors contributed to the article and approved the submitted version.

## Funding

This project is supported by Strategic Priority Research Program of the Chinese Academy of Sciences (Grant No. XDB27020105), National Science Foundation of China (31870214, 31701139), National Research and Development Program of Ministry of Science and Technology of China (2019YFA0904600, 2018YFA0900600). This project is funded also by Shanghai Sailing Program 17YF421900.

## Conflict of Interest

The authors declare that the research was conducted in the absence of any commercial or financial relationships that could be construed as a potential conflict of interest.

## Abbreviations

*A*, photosynthetic rate; AL, actinic light; CAT, catalase; CEF, cyclic electron flow; Chl, chlorophyll; *Ci*; internal CO_2_ concentration; d, days; DAB, 3, 3’ diaminobenzidine; DBMIB, 2,5-dibromo-3-methyl-6-isopropyl-p- benzoquinone; ETC, electron transport chain; FI, fluorescence induction; *gs*, stomatal conductance; F_o_, minimum fluorescence (PSII RCs open); F_m_, maximum fluorescence (PSII RCs close); *g*_ETC_, conductance of the electron transfer chain; *n*-PG, *n*-propyl gallate (*n*-PG, 3,4,5-trihydroxy-benzoic acid-n-propyl ester); NBT, nitroblue tetrazolium; NDH, NAD(P)H dehydrogenase; NDH-CET, NAD(P)H dehydrogenase-dependent cyclic electron transfer; NPQ, non-photochemical quenching; OJIP, Transient fast chlorophyll fluorescence induction with four steps called OJIP-curves; PPFD; Photosynthetic photon flux density; PQ, plastoquinone; PQH_2_, plastoquinol; PTOX, plastid terminal oxidase; P_700_, photosystem I reaction center; ROS, reactive oxygen species; SP, saturating pulse; SOD, superoxide dismutase; *SV*, *Setaria viridis*; *SA*, *Spartina alterniflora*.
