# Supplementary material for: Contrasting Responses of Plastid Terminal Oxidase Activity Under Salt Stress in Two C4 Species With Different Salt Tolerance
Source: Front Plant Sci. 2020 Jul 7;11:1009. doi: 10.3389/fpls.2020.01009 (PMC7359412; doi:10.3389/fpls.2020.01009)
Supplement: Supplementary file 1 [file DataSheet_1.docx]

| **Fluorescence parameters** | **Physiological significance** |
| --- | --- |
| F_0_ ≡ F_20µs_ | Minimal fluorescence when all the reaction centers are open |
| F_K_ ≡ F_300µs_ | Fluorescence intensity at 300 µs |
| F_J_ ≡ F_2ms_ | Fluorescence intensity at the J-step (2 ms) of OJIP |
| F_I_ ≡ F_30ms_ | Fluorescence intensity at the I-step (30 ms) of OJIP |
| F_m_ (≡ F_P_) | Maximal recorded fluorescence intensity, at the peak P of OJIP when all PSII reaction centers are closed |
| F_v_ = (F_m_-F_0_) | Variable chlorophyll fluorescence |
| t_Fm_ | Time to reach the maximal fluorescence intensity F_m_ |
| V_t_ = (F_t_ − F_0_)/(F_m_ − F_0_) | Relative variable fluorescence at time t |
| V_I_ = (F_I_ – F_0_)/(F_m_ – F_0_) | Relative variable fluorescence at the I-step |
| V_J_ = (F_J_ – F_0_)/(F_m_ – F_0_) | Relative variable fluorescence at the J-step |
| M_O_ = (∆V/∆t)_O_ = 4(F_300µs_ – F_0_)/(F_m_ – F_0_) | Approximated initial slope (in ms^-1^) of induction curve V_t_ (for F_0_ = F_20µs_) |
| Area | Integrated area between the induction curve and the line F=F_m_ relates to the pool size of PSII electron transport acceptors |
| S_M_ = Area/F_V_ | Normalized area (reflecting multiple turnover Q_A_ reduction events and representing energy necessary for the closure of all reaction centers |
| N = S_M_ M_O_ (1/V_J_) | Number of Q_A_ redox turn over until F_m_ is reached |
| ABS/RC = M_O_ (1/V_J_) (1/ᵠ_Po_) | Absorption flux (for PSII antenna chlorophylls) per reaction center (RC) |
| TR_O_/RC = M_O_ (1/V_J_) | Trapped energy flux (leading to Q_A_ reduction) per reaction center RC |
| ET_O_/RC = M_O_ (1/V_J_) ψ_o_ | Electron transport flux (further than Q_A_^–^) per PSII RC(at t = 0) |
| DI_O_/RC = (ABS/RC) - (TR_O_/RC) | Dissipated energy flux per reaction center RC (at t = 0) |
| ᵠ_Po_ = TR_O_/ABS = F_V_/F_m_ = [1 - (F_0_/F_m_)] | Maximum quantum yield of primary photochemistry (at t = 0) |
| ψ_o_ = ET_O_/TR_O_ = (1 - V_J_) | Probability that a traped exciton moves an electron further than Q_A_^–^ |
| ᵠEo = ET_O_/ABS = [1 - (F_0_/F_m_)] ψ_o_ | Probability that an absorbed photon moves an electron further than Q_A_^–^ |
| ABS/CSm = F_m_ (at t = t_Fm_) | Absorption flux per excited cross section, approximated by F_m_ |
| TR_O_/CSm = ᵠ_Po_ (ABS/CSm) (at t = t_Fm_) | Trapped energy flux per excited cross section, approximated by F_m_ |
| DI_O_/CSm = (ABS/CSm) - (TR_O_/CSm) (at t = t_Fm_) | Dissipated energy flux per excited cross section, approximated by F_m_ |
| ET_O_/CSm = ᵠ_Eo_ (ABS/CSm) (at t = t_Fm_) | Electron transport flux per excited cross section, approximated by F_m_ |
| RC/CSm = ψ_o_ (V_J_/M_O_)(ABS/CSm) | Density of reaction centers per excited cross-section (at t = tF_m_) |
| RC/ABS = [(F_2ms_ - F_0_)/4(F_300µs_ - F_0_)](F_V_/F_m_) | Density of reaction centers per chlorophyll |
| PI_(ABS)_ = (RC/ABS) (ᵠ_Po_/(1-ᵠ_Po_))-(ψ_o_/(1-ψ_o_)) | Performance index on absorption basis |
| PI_(CSm)_ = (RC/CSm) (ᵠ_Po_/(1-ᵠ_Po_))-(ψ_o_/(1-ψ_o_)) | Performance index on cross section basis |

**Table S1:** Definitions and explanations of selected JIP-test parameters used in the present study (modified from Strasser et al., 2004)

*** Sequences used for the synthesis of antibody raise against PTOX**

The synthesis of antibodies raised against PTOX in both species were designed using the protein sequence of PTOX for *Setaria viridis* and that of Maize for *Spartina alterniflora* based on the similarity degree of PTOX protein sequences (63%) between *Zea mays* and *Spartina alterniflora*, since the latter is not yet sequenced to date. So, we not have a complete available sequence for Spartina’s PTOX.

###

>GRMZM2G102349|GRMZM2G102349_T0|maize: CDS

ATGGCGGTGGCTTCGACCTCGCCGCTATCCGCCACGGCCCCCTCGCCGCCCGCTCCGGTGTCCGGGTTCCTCGCTCTCCCCGCCCGCCGCGGCTGCGCAACGCGCCTCGGCTCCGCCGCCGCGTGGAGGAGGCTTCGCGTGGAGGCGATCTGGAAGCAGCAGGAGAAGCAGCGGGCAGAGGTGTCCGTCGAGGAACCCGCCCCCGTCAGGGAGGCCGCCGCGCCCCTGGACGGAGTCGGAGCTGACGACCCCATGGTTCCTTCCTCGGACGAGAGCTGGGTGGTCAGGCTCGAGCAGTCGGTCAACATTTTCCTCACGGAATCGGTGATTATACTACTCAATACCGTGTACCGTGATCGGAACTACGCCAGGTTTTTTGTGCTGGAGACGATTGCCAGGGTGCCGTATTTCGCGTTCATATCGGTGCTTCACATGTATGAAACCTTTGGCTGGTGGAGACGAGCTGATTATCTAAAAGTTCACTTTGCGCAGAGCTTGAACGAGTTTCATCATCTCTTGATCATGGAAGAATTGGGTGGCAACGCTATATGGATTGATTGTTTCCTTGCTCGATTTATGGCGTTTTTTTACTACTTCATGACTGTTGCGATGTACATGTTGAGCCCACGAATGGCATATCACTTCTCTGAATGTGTGGAGAGACATGCGTACTCCACCTATGATAAGTTCCTCAAGCTCCATGAAGAGGAATTGAAAACACTACCAGCTCCAGAGGCAGCATTGAACTATTACCTGAATGAGGACCTTTACTTATTTGATGAGTTTCAGACAACAAGAATTCCATGTTCTAGGAGGCCTAAAATAGATAACTTGTATGATGTATTCGTCAATATACGAGATGACGAGGCAGAGCACTGCAAGACAATGAAGGCATGTCAAACACATGGAACTCTTCGTTCTCCTCACTCAATGCCGAACTGCTTAGAAGCTGCTACAGAATGTGTAATACCTGAAAACGATTGTGAAGGTATTGTGGACTGTGTCAAAAAGTCCCTTACAAAGTAA

###

>Spartina_PTOX|Contig2: cDNA

CTCACATCTCATTTCCACCCCACAAACGCCCACGAAACCGCAGCATGGCGGTGGCAGCCTCCACCTCCTCCTCCCCCCTCCCGGTCGCGTGCTCGCACCGGCGGGGCCCTTCCGGGTTCCTCCCGCTCCACGGCCACCGCGCCGCCACCGCCGCCACATGGAGGAGGAGGAGGCTCCACGCGGGGGCGATGAAGACGCAGCAGGAGAAGGAGCAGGCGGAGGCGACCGTCGAGGAGTCCTTCCCCGTGAGAGAGGCCGCGCCTTTGGGCGGAGCGGACGACCAGGCGGTTCCCACGGACGACAGCAGCTGGGTGGTGAATCTCGAGCAGTCCGTGAACATCTTCCTCGTGGATTCGGTGGTGACGATACTCGACAGTCTCTACCGTGACCGCAGTTACGCCAGGTTCTTTGTGTTGGAGACGATTGCTAGGGTGCCGTATTTCGCATTTATTTCCGTGCTTCACTTGTACGAGACCTTTGGCTGGTCGAGAAGAGCTGATTATATAAAGGTTCACTTTGCTGAGAGCATGAACGAGTTCCATCACCTCATGATCATGGAAGAACTGGGCGGCAACTCTGTATGGAGTGATCGTTTTCTGGCACGGTTTCTGGGCGTTTTTTTACTATTTCATGACTGTTGGGATGTACATGCTGAGCCCAAGAATGGCTTATCACTTTTCTGAATGCGTAGAGAGACATGCATACTCAACATATGACAAGTTTCTCAAGCTCAATGAAGAGGAGTTGAAAGGACTACCAGCTCCGGAGGCAGCTATAAACTATTATCTGAATGAGGATCTTTACTTATTTGACGAGTTTCAGACAGCAAGAGTTCCATGTTCAAGGAGGCCTAAAATCGATAACTTATACGATGTATTTATCAATGTACGAGATGATGAGGCGGAACATTGCAAGACAATGAAGGCCTGTCAAACAGATGGGAATCTTCAATCCCCGCACTCCACGAAGAGCTTAGAAATTGATACATAATGTGTAATACCTGCAAGCGATTGTGAAGTGTTCGGACATATGACATTGACATCACAAATACAATGTCCAATGGCGAAGTCAACGAATTCTATGATCAGGAGGCACAATGCTATCCGTCACCAGAGCTGTAGCAATGAGAGCACTGTCACCATGTAATGTCTTTCCACACAACTAGTGTAGAGGCAATCACCATTTCAACAAGCCTTTACATTTTTACCAGCTATCATCGGAACTGTTGTCATACCACCGTACGATCGTGTAACGTTAAGACCATCTGTGAATGTACCTATCTAAGCATATGCAGAAGTGTTATGTAGCAGAATAGACCAATCTTTTN

###

>Sevir.3G032600|Sevir.3G032600.1|seteria_viridis: CDS

ATGGCGGTGGCCTCCACCTCCATCCCGCCCCTCCCCGTGGCGCTGCCCGCCGCCGCCGCCGCCCGCTTCCTCCCGCTCCGCGGCCGCCGCGCCGCAGACCCGCGCCTCGGTCCCGTCGCCACGTGGAGGAGGTTCCGCGCGGAGGCGATTAAGACGCAGCGGGAGAAGCAGCAGACGGAGGTGCCCGTCGAGGAGTCCTTCCCCGCGAGGGAGGCCGCGCCGCTGGACGGAGCGGACGACCCGATGGTTCCATCGGATGAGGGCTGGGTGGTGAAGCTCGAGCAGTCGGTCAACATTTTGCTCACGGATTCGGTGATCATGGTACTCAATGGTGTTTACCGTGACCGGAGCTACGCCAGATTTTTTGTGCTGGAGACGATTGCTAGGGTGCCGTATTTTGCATTTATATCGGTGCTTCACTTGTATGAGACCTTTGGCTGGTCGAGGAGAGCTGATTATATAAAGGTTCACTTCGCTGAAAGCTGGAACGAGTTCCATCACCTCTTGATCATGGAAGAATTGGGTGGCAACGCTTTATTTTTTGATCGTTTCCTTGCTCGGTTCATGGCATTTTTTTACTACTTCATGACTGTTGGGATGTACATGCTGAGCCCAAGAATGGCATATCATTTTTCTGAATGTGTGGAGAGACATGCGTATTCAACTTATGACAAGTTTCTCAAGCTCCATGAAGAGGAGTTGAAAAGACTACCAGCTCCAGAGGCAGCTCTAAACTATTACCTGAACGAGGACCTTTACTTATTTGATGAGTTTCAGACAGCAAGAGTTCCATGTTCTAGGAGGCCTAAAATAGATAACTTGTATGATGTGTTTGTCAATATAAGAGACGATGAGGCAGAGCACTGCAAGACAATGAAGGCCTGTCAAACGCATGGAAGTCTTCGCTCTCCTCACTCAACACAGAACTGCTTAGAAGCTGATACGGAAGGTGTAATACCCGAAAAATGA
###
>GRMZM2G102349|GRMZM2G102349_T01

MAVASTSPLSATAPSPPAPVSGFLALPARRGCATRLGSAAAWRRLRVEAIWKQQEKQRAE
VSVEEPAPVREAAAPLDGVGADDPMVPSSDESWVVRLEQSVNIFLTESVIILLNTVYRDR
NYARFFVLETIARVPYFAFISVLHMYETFGWWRRADYLKVHFAQSLNEFHHLLIMEELGG
NAIWIDCFLARFMAFFYYFMTVAMYMLSPRMAYHFSECVERHAYSTYDKFLKLHEEELKT
LPAPEAALNYYLNEDLYLFDEFQTTRIPCSRRPKIDNLYDVFVNIRDDEAEHCKTMKACQ
THGTLRSPHSMPNCLEAATECVIPENDCEGIVDCVKKSLTK
###

> Spartina. Alterni protein seq-

MAVAASTSSSPLPVACSHRRGPSGFLPLHGHRAATAATWRRRRLHAGAMKTQQEKEQAEATVEESFPVREAAPLGGADDQAVPTDDSSWVVNLEQSVNIFLVDSVVTILDSLYRDRSYARFFVLETIARVPYFAFISVLHLYETFGWSRRADYIKVHFAESMNEFHHLMIMEELGGNSVWSDRFLARFLGVFLLFHDCWDVHAEPKNGLSLF

###

>Sevir.3G032600|Sevir.3G032600.1
MAVASTSIPPLPVALPAAAAARFLPLRGRRAADPRLGPVATWRRFRAEAIKTQREKQQTE
VPVEESFPAREAAPLDGADDPMVPSDEGWVVKLEQSVNILLTDSVIMVLNGVYRDRSYAR
FFVLETIARVPYFAFISVLHLYETFGWSRRADYIKVHFAESWNEFHHLLIMEELGGNALF
FDRFLARFMAFFYYFMTVGMYMLSPRMAYHFSECVERHAYSTYDKFLKLHEEELKRLPAP
EAALNYYLNEDLYLFDEFQTARVPCSRRPKIDNLYDVFVNIRDDEAEHCKTMKACQTHGS
LRSPHSTQNCLEADTEGVIPEK

###

* To design the RT-qPCR primers, we have used the PTOX Contig 6 of Spartina with degree of similarity was higher between protein sequences of maize and Spartina. It was around 74%. Results will be more accurate than using contig2 with 63% similarity.

*** Sequences used for the gene expression level of PTOX (q-RT-PCR)**

>Spartina_PTOX| Contig 2: CDS

Atggcggtggcagcctccacctcctcctcccccctcccggtcgcgtgctcgcaccggcggggcccttccgggttcctcccgctccacggccaccgcgccgccaccgccgccacatggaggaggaggaggctccacgcgggggcgatgaagacgcagcaggagaaggagcaggcggaggcgaccgtcgaggagtccttccccgtgagagaggccgcgcctttgggcggagcggacgaccaggcggttcccacggacgacagcagctgggtggtgaatctcgagcagtccgtgaacatcttcctcgtggattcggtggtgacgatactcgacagtctctaccgtgaccgcagttacgccaggttctttgtgttggagacgattgctagggtgccgtatttcgcatttatttccgtgcttcacttgtacgagacctttggctggtcgagaagagctgattatataaaggttcactttgctgagagcatgaacgagttccatcacctcatgatcatggaagaactgggcggcaactctgtatggagtgatcgttttctggcacggtttctgggcgtttttttactatttcatgactgttgggatgtacatgctgagcccaagaatggcttatcacttttc

###

>Spartina_PTOX| Contig6_protein-seq

MAVAASITSPLPSTLSANPTAARSHARAPPRSLPLHGHRVRAPRLGTVATWRRFRAEAMRTQQEKEQTEVAVEESFPAREAAPLDGADDQMVPTDDSWAVKLEQSVNVFLVDSVVTILDSFYRDRSYARFFVLETIARVPYFAFISVLHLYETFGWSRRADYIKVHFAESMNEFHHLLIMEELGGNSVWIDRFLARFLAFFYYFMTVGMYMLSPRMAYHFSECVERHAYSTYDKFLKLNEEELKGLPAPEVAINYYLNEDLYLFDEFQTARAPCSRRPKIDNLYDVFVNVRDDEAEHCKTMKACQTHGTLRSPHATKNNLETDT
###

>Spartina_PTOX| Contig6_CDS

atggcggtggcagcctccatcacctcccccctcccgtcgacgctctccgccaaccccaccgcggcgcgctcgcacgcgcgggcacctccccgatccctcccgctccacggccaccgcgtccgcgccccgcgcctcggcaccgtcgccacatggaggaggttccgcgcggaggcgatgaggacgcagcaggagaaggagcagacggaggtggccgtcgaggagtccttccccgcgagggaggccgcgcctttggacggagcggacgaccagatggttcccacggatgacagctgggcggtgaagctcgagcagtccgtgaacgttttccttgtggattcggtggtgacgatattagacagtttctaccgtgaccgcagttacgccaggttttttgtattggagacgattgccagggtgccgtatttcgcatttatatcggtgcttcacttgtatgagaccttcggctggtccagaagagctgattatataaaggttcacttcgctgagagcatgaacgagttccatcacctcttgatcatggaagaattgggcggcaactctgtatggatcgatcgttttcttgcacgatttctggcgtttttttactatttcatgactgttgggatgtacatgctgagcccaagaatggcttatcacttttctgaatgcgtagagagacacgcatactcaacttatgacaagtttctcaagctcaatgaagaggagttgaaagggctaccagctccagaggtagctataaactattatttgaatgaagatctttacttatttgacgagtttcagacagcaagagctccatgctcaagaaggcctaaaattgataacttatacgatgtatttgtcaatgtacgagatgacgaggcggaacactgcaagacaatgaaggcctgtcaaacacatggaacacttcgctcccctcacgccacaaagaacaacttagagaccgataca

**Reference gene (housekeeping) for Spartina and Setaria: Tubulin alpha**

CDS sequence

>Sevir.9G071900|Sevir.9G071900.1; Setaria_tubulin_alpha

ATGAGGGAGTGCATCTCGATCCACATCGGGCAGGCCGGCATCCAGGTCGGCAACGCGTGCTGGGAGCTCTACTGCCTCGAGCACGGCATCCAGCCTGATGGCCACATGCCCGGAGACAAGTCTGCAGGACACTACGATGATGCCTTCACCACCTTCTTCAGCCAGACCGGCGCAGGGAAGTATGTGCCCCGTGCAATCTTCGTTGATCTTGAGCCCACTGTGATTGATGAGGTGCGCACCGGCATATACCGTCAGCTCTTCCACCCTGAGCAGCTCATCAGCGGCAAGGAGGATGCAGCCAACAACTTTGCTCGTGGCCACTACACAATTGGCAAGGAGATTGTTGATCTGTGCCTTGACCGCATCCGCAAGCTTGCTGACAACTGCACTGGCCTTCAGGGCTTTCTGGTCTTCAATGCTGTTGGTGGTGGCACCGGTTCTGGCCTTGGTTCACTCCTCCTTGAGCGCCTGTCTGTGGACTACGGCAAGAAATCCAAACTGGGCTTCACTGTGTACCCATCTCCCCAGGTGTCAACCTCTGTTGTTGAGCCCTACAACAGCGTGCTCTCCACCCACTCTCTTCTGGAGCATACTGATGTCTCCATCCTGCTCGACAACGAGGCCATCTATGACATCTGCAGGCGCTCTCTGGACATTGAGAGGCCCAACTACTCCAATCTGAATCGCCTTGTGTCTCAGGTTATCTCATCGCTGACTGCTTCCCTGAGGTTTGATGGTGCCCTCAATGTGGATGTGAATGAGTTCCAGACCAACCTGGTTCCTTACCCAAGGATCCACTTCATGCTGTCCTCGTATGCGCCGGTGATCTCTTCAGAGAAGGCCTACCATGAGCAGCTGTCGGTGTCGGAGATCACCAACAGCGCGTTCGAGCCGGCGAACATGATGGTCAAGTGTGACCCCCGCCACGGCAAGTACATGGCGTGCTGCCTGATGTACCGCGGCGATGTGGTGCCCAAGGATGTGAACGCGGCGGTGGCCACCATCAAGACGAAGCGCACGATCCAGTTTGTGGACTGGTGCCCAACAGGGTTCAAGTGCGGCATCAACTACCAGGCACCGACAGTGGTGCCGGGTGGTGACCTCGCCAAGGTGCAGCGCGCTGTGTGCATGATCTCCAACTCCACCAGCGTCGCTGAGGTGTTCTCCCGCATCGACCGCAAGTTCGACCTCATGTATGCCAAGCGCGCCTTTGTGCACTGGTATGTCGGCGAGGGCATGGAGGAGGGGGAGTTCTCCGAGGCCCGTGAGGACCTGGCGGCCCTGGAGAAGGACTACGAGGAGGTCGGCGCTGAGGGCGGTGGTGACGATGATGAGGAGGACGAGGAGTACTGA

>Contig15_spartina_tubulin_alpha

atgagggagtgcatctcgatccacatcggccaggccggtatccaggtcggaaacgcgtgctgggagctgtactgcctcgaacatggcattcaggctgacggtcagatgcctggtgacaagaccattggaggaggtgatgatgctttcaacaccttcttcagtgagactggcgctggcaagcatgtgccccgtgccgtgtttgttgaccttgagcccactgtgattgatgaggtgaggactggcacttaccgccagctcttccaccctgagcagctcatcagtggtaaggaggatgcagccaacaactttgcccgtggtcactacaccattggcaaggagattgttgacctgtgccttgaccgcatcaggaagcttgccgacaactgtactggtctccagggtttccttgtcttcaatgctgtcggtggaggaactggctctggtcttggttccctcctccttgagcgtctgtctgttgactatggcaagaagtccaagctcgggttcactgtgtacccatcaccccaggtctccacctcggtggttgagccatacaacagtgtcctgtccactcactcgctcctcgagcacactgatgttgctgtgctgcttgacaatgaggccatctacgacatctgccgccgctccctcgacattgagcgcccaacctacaccaatctcaacaggcttgtatctcaggtcatctcatctctgacagcctccctgaggtttgatggtgctctgaacgtggatgtgaacgagttccagaccaacctggtgccctaccccaggatccacttcatgctttcgtcctacgcgccagtgatctctgccgagaaggcctaccacgagcagctgtctgttgctgagatcaccaacagtgccttcgaaccttcctccatgatggctaagtgtgacccccgccatggcaagtacatggcttgctgcctcatgtaccgtggtgatgttgtgcccaaggacgtgaacgctgctgtggccaccatcaagaccaagcgcaccattcagttcgtggactggtgccccactggcttcaagtgcggtatcaactaccagccacccagcgttgtccctggtggcgacctggccaaggtgcagagggccgtgtgcatgatctccaactccaccagtgttgtggaggtgttctcacgcatcgaccacaagttcgacctcatgtacgccaagcgtgcctttgccacgtggtacgtgggtgagggcagtgggaggagggcg

Tubulin alpha gene

Tub-F 5' AGGGAGTGCATCTCGATCCAC 3'

Tub-R 5' ACCTCATCAATCACAGTGGGCTC 3'

Product 230bp

Similarity 85%

**PTOX sequence similarity: 73%**

**>Sevir.3G032600|Sevir.3G032600.1|Seteria_viridis: CDS**

ATGGCGGTGGCCTCCACCTCCATCCCGCCCCTCCCCGTGGCGCTGCCCGCCGCCGCCGCCGCCCGCTTCCTCCCGCTCCGCGGCCGCCGCGCCGCAGACCCGCGCCTCGGTCCCGTCGCCACGTGGAGGAGGTTCCGCGCGGAGGCGATTAAGACGCAGCGGGAGAAGCAGCAGACGGAGGTGCCCGTCGAGGAGTCCTTCCCCGCGAGGGAGGCCGCGCCGCTGGACGGAGCGGACGACCCGATGGTTCCATCGGATGAGGGCTGGGTGGTGAAGCTCGAGCAGTCGGTCAACATTTTGCTCACGGATTCGGTGATCATGGTACTCAATGGTGTTTACCGTGACCGGAGCTACGCCAGATTTTTTGTGCTGGAGACGATTGCTAGGGTGCCGTATTTTGCATTTATATCGGTGCTTCACTTGTATGAGACCTTTGGCTGGTCGAGGAGAGCTGATTATATAAAGGTTCACTTCGCTGAAAGCTGGAACGAGTTCCATCACCTCTTGATCATGGAAGAATTGGGTGGCAACGCTTTATTTTTTGATCGTTTCCTTGCTCGGTTCATGGCATTTTTTTACTACTTCATGACTGTTGGGATGTACATGCTGAGCCCAAGAATGGCATATCATTTTTCTGAATGTGTGGAGAGACATGCGTATTCAACTTATGACAAGTTTCTCAAGCTCCATGAAGAGGAGTTGAAAAGACTACCAGCTCCAGAGGCAGCTCTAAACTATTACCTGAACGAGGACCTTTACTTATTTGATGAGTTTCAGACAGCAAGAGTTCCATGTTCTAGGAGGCCTAAAATAGATAACTTGTATGATGTGTTTGTCAATATAAGAGACGATGAGGCAGAGCACTGCAAGACAATGAAGGCCTGTCAAACGCATGGAAGTCTTCGCTCTCCTCACTCAACACAGAACTGCTTAGAAGCTGATACGGAAGGTGTAATACCCGAAAAATGA

**PTOX_SV_-F** 5' CGATTGCTAGGGTGCCGTAT 3'

**PTOX_SV_-R** 5' AAAGCGTTGCCACCCAATTC 3'

**Product length: 211**

**>Spartina_PTOX| Contig 6_CDS**

atggcggtggcagcctccatcacctcccccctcccgtcgacgctctccgccaaccccaccgcggcgcgctcgcacgcgcgggcacctccccgatccctcccgctccacggccaccgcgtccgcgccccgcgcctcggcaccgtcgccacatggaggaggttccgcgcggaggcgatgaggacgcagcaggagaaggagcagacggaggtggccgtcgaggagtccttccccgcgagggaggccgcgcctttggacggagcggacgaccagatggttcccacggatgacagctgggcggtgaagctcgagcagtccgtgaacgttttccttgtggattcggtggtgacgatattagacagtttctaccgtgaccgcagttacgccaggttttttgtattggagacgattgccagggtgccgtatttcgcatttatatcggtgcttcacttgtatgagaccttcggctggtccagaagagctgattatataaaggttcacttcgctgagagcatgaacgagttccatcacctcttgatcatggaagaattgggcggcaactctgtatggatcgatcgttttcttgcacgatttctggcgtttttttactatttcatgactgttgggatgtacatgctgagcccaagaatggcttatcacttttctgaatgcgtagagagacacgcatactcaacttatgacaagtttctcaagctcaatgaagaggagttgaaagggctaccagctccagaggtagctataaactattatttgaatgaagatctttacttatttgacgagtttcagacagcaagagctccatgctcaagaaggcctaaaattgataacttatacgatgtatttgtcaatgtacgagatgacgaggcggaacactgcaagacaatgaaggcctgtcaaacacatggaacacttcgctcccctcacgccacaaagaacaacttagagaccgataca

**PTOX_SA_ F** 5’ CCTTGTGGATTCGGTGGTGA 3’

**PTOX_SA_ R** 5’ GCTCTCAGCGAAGTGAACCT 3’

**Product length 184**

**Table S2:** Primers list used to determine the PTOX expression level with qPCR analysis relative to the reference gene Tubuline-alpha (Tub) according to Livak and Schmittgen (2001) formula: 2^−ΔΔCT^ (ΔCT = CT, gene of interest−CT, Tubulin-alpha).

| **Gene** | **Gene name** | **Primers** | **Product size** |
| --- | --- | --- | --- |
| Tub-F | Tubulin alpha | AGGGAGTGCATCTCGATCCAC | 230 |
| Tub-R | Tubulin alpha | ACCTCATCAATCACAGTGGGCTC | - |
| PTOX_SV_-F | PTOX | CGATTGCTAGGGTGCCGTAT | 211 |
| PTOX_SV_-R | PTOX | AAAGCGTTGCCACCCAATTC | - |
| PTOX_SA_-F | PTOX | CCTTGTGGATTCGGTGGTGA | 184 |
| PTOX_SA_-R | PTOX | GCTCTCAGCGAAGTGAACCT | - |
